# What does fluorine do to a protein? Thermodynamic, and highly-resolved structural insights into fluorine-labelled variants of the cold shock protein

**DOI:** 10.1038/s41598-020-59446-w

**Published:** 2020-02-14

**Authors:** Hannah Welte, Tiankun Zhou, Xenia Mihajlenko, Olga Mayans, Michael Kovermann

**Affiliations:** 1 Department of Chemistry, Universitätsstrasse 10, Universität Konstanz, DE-78457 Konstanz, Germany; 2 Graduate School Chemical Biology KoRS-CB, Universitätsstrasse 10, Universität Konstanz, DE-78457 Konstanz, Germany; 3 Department of Biology, Universitätsstrasse 10, Universität Konstanz, DE-78457 Konstanz, Germany; 4 Zukunftskolleg, Universitätsstrasse 10, Universität Konstanz, DE-78457 Konstanz, Germany

**Keywords:** Biophysical chemistry, X-ray crystallography, NMR spectroscopy

## Abstract

Fluorine labelling represents one promising approach to study proteins in their native environment due to efficient suppressing of background signals. Here, we systematically probe inherent thermodynamic and structural characteristics of the Cold shock protein B from *Bacillus subtilis* (*Bs*CspB) upon fluorine labelling. A sophisticated combination of fluorescence and NMR experiments has been applied to elucidate potential perturbations due to insertion of fluorine into the protein. We show that single fluorine labelling of phenylalanine or tryptophan residues has neither significant impact on thermodynamic stability nor on folding kinetics compared to wild type *Bs*CspB. Structure determination of fluorinated phenylalanine and tryptophan labelled *Bs*CspB using X-ray crystallography reveals no displacements even for the orientation of fluorinated aromatic side chains in comparison to wild type *Bs*CspB. Hence we propose that single fluorinated phenylalanine and tryptophan residues used for protein labelling may serve as ideal probes to reliably characterize inherent features of proteins that are present in a highly biological context like the cell.

## Introduction

Proteins are key operating elements of complex biological systems, such as cells. These macromolecules control a multiplicity of chemical processes and are a central characteristic of living systems^1^. The determination of the three-dimensional structure of a protein and the identification of its potential interacting partners is crucial to understand the function of these macromolecules in biological systems. However, most studies aiming to unravel the intracellular functionality of proteins are performed *in vitro*, using dilute experimental conditions that are distant from the crowded cellular environments that proteins experience in living organisms. The total intracellular macromolecular concentration can be estimated with up to about 400 g/l^2,3^ whereas individual proteins are often present in tiny amounts. Thus, performing protein studies using *in vitro* conditions neglects the intracellular environment and does not account for a variety of effects like e.g. crowding^4^, confinement^5^ or quinary interactions^6^. In the recent years, significant progress has been made on the study of proteins in native cellular environments. In this regard, high-resolution NMR spectroscopy is a promising technique that offers indispensable insights into the structure, dynamics, and stability of the protein under study in an intracellular context^7–9^. The specific labelling of proteins using fluorine^10,11^ may represent a successful strategy to perform *in cellula* NMR experiments. Once the protein has been successfully labelled using fluorine, the determination of kinetic and thermodynamic parameters (such as the change in enthalpy, *ΔH*, the change in heat capacity, *ΔC*_P_, upon protein unfolding, and the folding and unfolding rate constants) is possible even using cell-like conditions^12–14^. Thus, the labelling of proteins using fluorine allows conducting a broad variety of NMR spectroscopic experiments focusing on ^19^F resonances. This approach offers several advantages and simplifications compared to experiments commonly used in biomolecular NMR spectroscopy that focus on ^1^H, ^13^C, and ^15^N nuclei^14,15^. In this respect, fluorine has a natural abundance of 100%, a high sensitivity (83% compared to ^1^H), a large range of chemical shifts and no natural occurrence in proteins enabling to efficiently suppress undesired background signals. Thereby, structural, dynamical, and functional protein information can be obtained at atomic resolution in complex biological environments, even if the protein of interest is only present in a relatively low concentration^12,15,16^. We do not conceal that fluorine possesses large chemical shift anisotropy^17^ which may potentially impede assignment and interpretation of fluorine NMR spectra. However, as leading examples of the applicability of ^19^F-derivatized proteins, fluorotryptophan has been incorporated into the carbohydrate binding protein lectin from *Ralstonia solanacearum* enabling monitoring its interaction with ligands at atomic detail by X-ray crystallography^18^, whereas ^19^F NMR spectroscopy has been successfully applied to reveal the exchange dynamics present in antibody-antigen binding, an information that is not accessible using X-ray crystallography^19^. Furthermore, selective fluorine-labelling has been successfully used to derive distance restraints for a protein based on ^19^F paramagnetic relaxation enhancement experiments^20^ whereas the interaction sites present in protein-ligand complexes have been elucidated by applying ^19^F pseudocontact shift analysis^21^. An elegant combination of X-ray crystallography, NMR spectroscopy, and MD simulations has been recently applied to unravel the distribution of conformational states along the reaction pathway of a homodimeric enzyme^22^. Pomerantz and co-workers have successfully utilized fluoroaromatic amino acids for the implementation into proteins to report on the structure-activity relationship^23^ focusing on screening of small molecule-protein interactions based on protein-observed fluorine NMR spectroscopy^24^. Moreover, incorporation of difluorotyrosine has been successfully employed enabling to report on tyrosine phosphorylation^25^ or to probe distinct conformational states of a protein which are related to signalling by applying ^19^F NMR spectroscopy^26^. In a recent study, an NMR based comparison of the incorporation of 2- and 3-fluorotyrosine into a KIX domain has been presented^27^.

The outstanding question in the application of ^19^F-NMR based methodologies is how much does the ^19^F-modification impact the inherent properties of the protein under study, particularly its atomic three-dimensional structure, conformational dynamics, and its overall thermodynamic stability. Addressing this question is of high importance as the fluorine-labelled protein variant, and not the wild type, is used in *in cellula* NMR spectroscopy to report on the native structural and dynamical features of proteins *in vivo*. In this context, it has been shown that extensively fluorinated amino acids can be particularly effective in increasing protein stability^28^ due to the increase in buried hydrophobic surface area as identified in structures solved by X-ray crystallography^29^. Kitevski-LeBlanc and co-workers have shown that ^19^F enrichment in fluoro-phenyalanine labelled calmodulin results in an increasing protein disorder which can be diminished by a decreased level of fluorination^30^. Other work has shown that the structural integrity of a small single domain protein is conserved when one fluoro-phenylalanine is incorporated^31^ and that fluoro-tryptophan labelling of various sites in fluoroacetate dehalogenase does not modify its three-dimensional structural characteristics compared to the wild type^22^.

**Figure 1:**
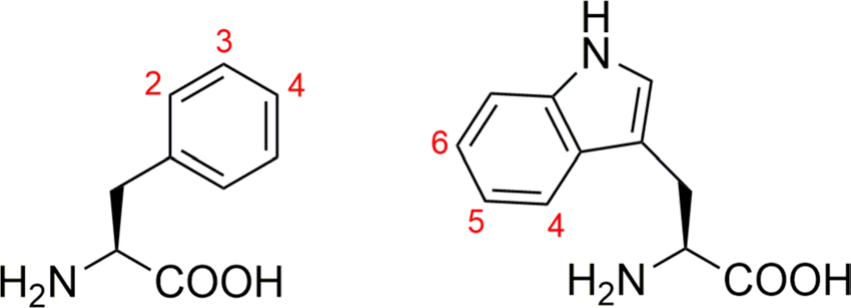
Numbering of sites used for single fluorine labelling in 2-^19^F-, 3-^19^F-, and 4-^19^F-phenylalanine (left) and 4-^19^F-, 5-^19^F-, and 6-^19^F-tryptophan (right). The structures have been created by using ChemDraw18 (www.perkinelmer.com).

In previous work, we have used auxotrophic *E. coli* strains to incorporate fluorinated phenylalanine (Phe) or tryptophan (Trp) amino acid residues into the Cold shock protein B from *Bacillus subtilis* (*Bs*CspB). Within these residues, we used different sites for the incorporation of the fluorine atoms, generating a total of three Phe and three Trp modified amino acids (Fig. 1)^32^. Using this tool kit, we were then able to successfully prepare six different ^19^F-labelled *Bs*CspB variants (2-^19^F-Phe-, 3-^19^F-Phe-, 4-^19^F-Phe-, 4-^19^F-Trp-, 5-^19^F-Trp- and 6-^19^F-Trp-*Bs*CpB) at a milligram scale and in high purity enabling high resolution one-dimensional ^1^H, ^19^F and two-dimensional ^1^H-^15^N correlation NMR spectroscopy of the samples mentioned above^32^. *Bs*CspB is a relatively small protein of 67 amino acids length that belongs to the cold shock protein family^33–35^. It folds into five beta-strands that form a beta-barrel structure^36,37^, which binds single-stranded DNA and RNA^38,39^. Notably, *Bs*CspB shows an overall thermodynamic stability of about *ΔG*° = 10 kJ mol^−1^ at *T* = 298 K and possesses fast unfolding, *k*_u_, and refolding, *k*_f_, rate constants of about *k*_u_ = 12 s^−1^ and *k*_f_ = 1070 s^−1^, respectively^33^. Here, we focus on the precise determination of thermodynamic and kinetic parameters of all six fluorine-labelled protein variants in comparison to wild type *Bs*CspB. A combination of fluorescence, kinetic stopped flow, NMR spectroscopy, and X-ray crystallography has been applied to obtain an integrated understanding of potential effects of fluorine-labelling in proteins. Our findings show that fluorine-labelling of proteins utilizing singly modified amino acids like Phe or Trp does not cause a detectable alteration of thermodynamic and structural properties of *Bs*CspB. The present study closes an important gap in the basic characterization of fluorine-labelled proteins and underlines that this methodology may serve as an optimal tool to study proteins in their native complex biological environment applying high-resolution NMR spectroscopy.

## Results and Discussion

### Thermodynamic stability probed by fluorescence in equilibrium

**Figure 2:**
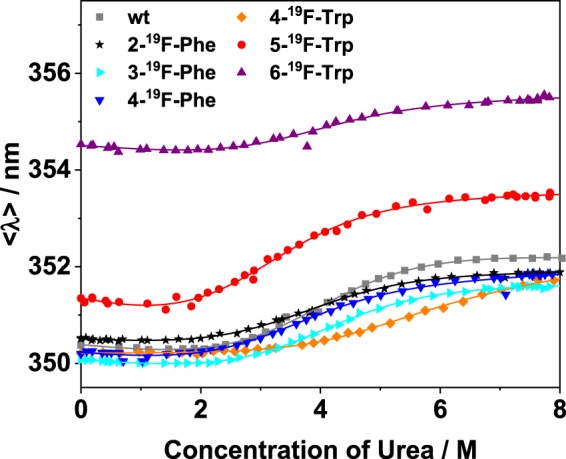
Dependence of average emission wavelength on the concentration of urea obtained from fluorescence spectra for all protein variants probed in this study (rectangles represent wild type protein, stars represent 2-^19^F-Phe-, triangles with the tip to right represent 3-^19^F-Phe-, triangles with the tip to bottom represent 4-^19^F-Phe-, diamonds represent 4-^19^F-Trp-, circles represent 5-^19^F-Trp-, and triangles with the tip to top represent 6-^19^F-Trp-variant). The straight lines represent best fits of Eq. (3) to the experimental data sharing the same value for the cooperativity of unfolding, *m*. Results of this fitting procedure are summarized in Table 1.

Intrinsic fluorescence spectroscopy has been applied to probe the overall thermodynamic stability of the six differently fluorine-labelled variants of *Bs*CspB. For this purpose, folded-to-unfolding transitions of *Bs*CspB samples were chemically induced with increasing amounts of urea and monitored using fluorescence spectroscopy in equilibrium. Specifically, fluorescence emission spectra of wild type and 2-^19^F-Phe-, 3-^19^F-Phe-, 4-^19^F-Phe-, 4-^19^F-Trp-, 5-^19^F-Trp-, and 6-^19^F-Trp-labelled *Bs*CspB were measured in urea concentrations ranging from *c*_urea_ = 0 M to *c*_urea_ = 8 M. Note that all seven intrinsic Phe residues Phe9, Phe15, Phe17, Phe27, Phe30, Phe38, and Phe45 present in *Bs*CspB have been equally fluorine-labelled in 2-^19^F-Phe-, 3-^19^F-Phe- or 4-^19^F-Phe-modified protein samples^32^ (Fig. S1A) whereas 4-^19^F-Trp-, 5-^19^F-Trp-, and 6-^19^F-Trp-*Bs*CspB possess Trp8 as single site of the modification (Fig. S1B). Fluorescence spectra showed that, upon unfolding, the maximal fluorescence emission intensity decreased (Figs. S2A–S8A) and shifted to significantly larger wavelengths (Figs. S2B–S7B) for both wild type and fluorine-labelled variants. The signal shifting to larger wavelengths regarding fluorescence emission of tryptophans is consistent with the aromatic group becoming more exposed to the polar solvent upon unfolding of the protein. The properties observed here for all six differently fluorine-labelled variants of *Bs*CspB regarding fluorescence emission upon chemical unfolding have been also reported for the wild type^33^. Solely 6-^19^F-Trp-*Bs*CspB does not show a dependence of the maximum wavelength in fluorescence emission, 𝜆^max^, on the concentration of urea (Fig. S8B) but preserving a decrease in fluorescence emission intensity by an increasing concentration of urea (Fig. S8A). The lack in the increase of the maximum wavelength in fluorescence emission observed for the 6-^19^F-Trp-labelled variant can be anticipated as 𝜆^max^ is about 360 nm already in the absence of urea (Fig. S8B). However, 𝜆^max^ observed for 6-^19^F-Trp-*Bs*CspB has been also approached for remaining variants of *Bs*CspB at high concentrations of urea (Figs. S2B–S7B). The fluorescence emission intensity is lowest for 4-^19^F-Trp-*Bs*CspB among all probed fluorine-labelled protein variants (Fig. S6A). This observation is based on the inherent low fluorescence quantum yield of 4-^19^F-Trp^40^ which is about 100 times lower compared to Trp, 5-^19^F-Trp, and 6-^19^F-Trp (Fig. S9). For fluorescence data analysis the intensity emission averaged wavelength, < 𝜆 >, has been determined (Fig. 2). This procedure is based on two rationales. Firstly, the maximum of fluorescence emission of *Bs*CspB variants shifts by an increasing concentration of urea to larger wavelengths (see above). Secondly, the maximum of fluorescence emission is dependent on the site used for inserting fluorine into free tryptophan residues (Fig. S9). Thus the free form of native Trp shows a maximum in fluorescence emission at 𝜆^max^ = 353 nm, free 5-^19^F-Trp at 𝜆^max^ = 360 nm, free 6-^19^F-Trp at 𝜆^max^ = 360 nm, and free 4-^19^F-Trp 𝜆^max^ = 376 nm using 𝜆 = 280 nm for excitation going along with observations on free fluorotryptophans performed before using 𝜆 = 295 nm for excitation^40^.

The intensity averaged emission wavelength < λ > is calculated using Eq. (1)

1 $$ < \lambda  > =\mathop{\sum }\limits_{i=320.5\,{\rm{nm}}}^{380\,{\rm{nm}}}({I}_{i}{\lambda }_{i})/\mathop{\sum }\limits_{i=320.5\,{\rm{nm}}}^{380\,{\rm{nm}}}({I}_{i}),$$

where *I*_i_ is the intensity of fluorescence emission at wavelength λ_i_^41^.

This method of fluorescence data analysis accounts for both potential changes in the shape of acquired fluorescence emission spectra and a potential shift in wavelength of the maximum in fluorescence emission, respectively. Note that measuring the refractive index of the buffer accounts for the precise determination of the concentration of urea which has been individually used (Eq. 2).

Applying a two state folding-to-unfolding model^42^ (Eq. 3) to the experimentally obtained fluorescence data yields the free energy against unfolding, *ΔG*°, of wild type *Bs*CspB and all six variants differing in the site used for fluorine labelling. The cooperativity of protein unfolding, *m*, is then used as a global fitting parameter enabling a direct comparison of the overall thermodynamic stability of all seven different protein variants probed in the present study. This approach results in *m* = −2.9 ± 0.1 kJ/(mol M), which agrees well with *m* values which have been reported for *Bs*CspB before, *m* = −3.2 ± 0.1 kJ/(mol M)^33,43^. The free energy of protein unfolding ranges between *ΔG*° = 9.0 ± 0.5 kJ/mol obtained for 5-^19^F-Trp-BsCspB and *ΔG*° = 12.7 ± 1.1 kJ/mol for 4-^19^F-Trp-*Bs*CspB (Table 1). Thus, the overall thermodynamic stability of fluorine-labelled *Bs*CspB variants reproduces the stability of wild type *Bs*CspB, determined here using the same type of data analysis (Δ*G*° = 11.1 ± 0.5 kJ/mol) (Table 1). Note that linear fitting of fluorescence emission intensities characterizing the transition region between folded and unfolded state has been also performed for all variants of *Bs*CspB^44^ (Fig. S10, Table S1). This procedure ensures that the determination of the overall thermodynamic stability *ΔG*° is not primarily influenced by missing baselines representing the folded as well as the unfolded state as it has been shown by Alexander and Pace^44^. Notably, both approaches used for the analysis of fluorescence emission intensities yield consistent results for *ΔG*° (Table S1).

**Table 1:** Analysis of equilibrium unfolding of wild type and fluorine-labelled variants of *Bs*CspB using *fluorescence spectroscopy* (Fig. 2) to determine the overall thermodynamic stability, Δ*G*^0^. The cooperativity of protein unfolding has been used as global parameter in Eq. (3) taking all seven folded-to-unfolding transitions into account and has been determined to *m* = −2.9 ± 0.1 kJ/(mol M).

Protein	Δ*G*^0^/kJ/mol	*C*_M_/M
wt *Bs*CspB	11.1 ± 0.5	3.8 ± 0.2
2-^19^F-Phe-*Bs*CspB	10.0 ± 0.7	3.5 ± 0.2
3-^19^F-Phe-*Bs*CspB	10.5 ± 0.6	3.6 ± 0.2
4-^19^F-Phe-*Bs*CspB	9.6 ± 0.6	3.3 ± 0.2
4-^19^F-Trp-*Bs*CspB	12.7 ± 1.1	4.4 ± 0.4
5-^19^F-Trp-*Bs*CspB	9.0 ± 0.5	3.1 ± 0.2
6-^19^F-Trp-*Bs*CspB	11.0 ± 0.7	3.8 ± 0.2

The midpoint of folded-to-unfolding transition, *C*_M_, has been calculated using *C*_M_ = Δ*G*^0^/*m*.

Summing up, fluorine labelling of either tryptophan or phenylalanine residues does not have a major effect on the overall thermodynamic stability of cold shock protein B when monitoring fluorescence emission dependent on an increasing concentration of urea. The overall thermodynamic stability determined for 4-^19^F-Trp-*Bs*CspB is highest comparing all variants probed in the present study (Table 1) and deviates in *ΔG*° by 1.6 kJ/mol from wild type *Bs*CspB. This property of 4-^19^F-Trp-*Bs*CspB has been one rationale to elucidate the structural features of this fluorine-labelled variant of *Bs*CspB by using X-ray crystallography in more detail. Focusing on phenylalanine modified variants, 4-^19^F-Phe-*Bs*CspB deviates most in *ΔG*° from wild type *Bs*CspB by 1.5 kJ/mol. This property of 4-^19^F-Phe-*Bs*CspB has been one rationale for the crystallization of this variant enabling to obtain atomically resolved information (see below).

### Thermodynamic stability probed by NMR spectroscopy

**Figure 3:**
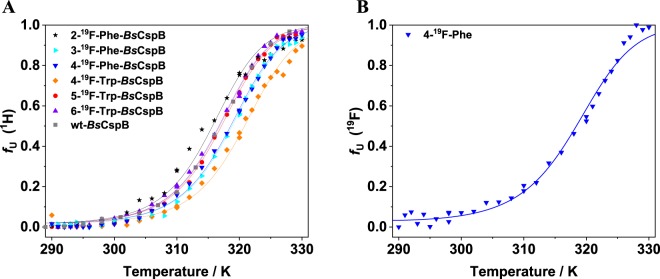
Folded-to-unfolding transitions monitored by using one-dimensional ^1^H (**A**) and ^19^F (**B**) NMR spectroscopy. The thermal denaturation has been probed for wild type (rectangles), 2-^19^F-Phe- (stars), 3-^19^F-Phe- (triangles with the tip to right), 4-^19^F-Phe- (triangles with the tip to bottom), 4-^19^F-Trp- (diamonds), 5-^19^F-Trp- (circles), and 6-^19^F-Trp-variant (triangles with the tip to top). The straight lines represent best fits of Eq. (4) to the experimental data sharing the same value for the change in enthalpy, *ΔH*. Results of this fitting procedure are summarized in Table 2.

The characterization of the thermodynamic stability of differently fluorine-labelled variants of *Bs*CspB has been expanded to folded-to-unfolding transitions induced by increasing the temperature. The thermal denaturation of the six ^19^F-labelled *Bs*CspB variants and wild type *Bs*CspB has been monitored by one-dimensional ^1^H (Fig. S11) and ^19^F NMR spectroscopy (holds for 4-^19^F-Phe-*Bs*CspB) to independently verify the experimental results obtained for chemical denaturation monitored using intrinsic fluorescence emission (see above). Advantageously, NMR spectroscopy provides direct spectroscopic information about the folded-to-unfolding transition of the entire protein under investigation whereas fluorescence spectroscopy operates as a local reporter of aromatic residues only. Thus, one-dimensional ^1^H and ^19^F NMR spectra have been acquired for different variants of *Bs*CspB including the wild type in the temperature range *T* = 291 K to *T* = 330 K (Figs. S11A,B–S17A,B). The fitting of Eq. (4) to the experimentally obtained data representing the fraction of unfolded protein gives access to the change in heat capacity, *Δ*C_P_, the change in enthalpy, *ΔH*, and the temperature midpoint characterizing the folded-to-unfolding transition, *T*_M_, for all variants of *Bs*CspB which have been probed here. Note that the change in heat capacity has been fixed to *ΔC*_P_ = 5.8 kJ/(mol K) here as it has been specifically determined for *Bs*CspB using ^1^H NMR spectroscopy before relying on both heat and cold denaturing^45^. The change in enthalpy has been used as a global parameter for all variants of *Bs*CspB when fitting Eq. (4) to the experimental data acquired by one-dimensional ^1^H spectroscopy whereas *T*_M_ has been individually determined for all variants (Fig. 3A, Table 2). This procedure leads to Δ*H* = 197 ± 2 kJ/mol and values for *T*_M_ ranging between *T*_M_ = 315.6 K (2-^19^F-Phe-*Bs*CspB) and *T*_M_ = 320.8 K (4-^19^F-Trp-*Bs*CspB). Note that wild type *Bs*CspB shows *T*_M_ = 316.8 K indicating that there exists no significant difference in thermodynamic stability to the variously fluorine-labelled variants of *Bs*CspB. This result independently verifies the observation made for the chemical denaturation of *Bs*CspB variants presented before. The fitting of Eq. (4) to the data obtained for 4-^19^F-Phe-*Bs*CspB using one-dimensional ^19^F NMR spectroscopy yield *T*_M_ = 318.4 K and Δ*H* = 176 ± 6 kJ/mol (Fig. 3B) confirming the thermodynamic analysis done by using ^1^H NMR spectroscopic data which has illuminated *T*_M_ = 318.5 K (Table 2). Note that a precise thermodynamic analysis of ^19^F NMR detected folding-to-unfolding transitions of 2-^19^F-Phe-, 3-^19^F-Phe-, 4-^19^F-Trp-, 5-^19^F-Trp-, and 6-^19^F-Trp-*Bs*CspB has been prohibited due to spectral indistinguishability of signals representing either the native state or the unfolded ensemble of the protein under study.

**Table 2:** Analysis of equilibrium unfolding of wild type and fluorine-labelled variants of *Bs*CspB using one-dimensional ^1^H and ^19^F *NMR spectroscopy* to determine the midpoint of folded-to-unfolding transition, *T*_M_.

Protein	*T*_M_/K
wt *Bs*CspB	316.8 ± 0.2
2-^19^F-Phe-*Bs*CspB	315.6 ± 0.1
3-^19^F-Phe-*Bs*CspB	318.7 ± 0.1
4-^19^F-Phe-*Bs*CspB	318.5 ± 0.1
4-^19^F-Trp-*Bs*CspB	320.8 ± 0.1
5-^19^F-Trp-*Bs*CspB	317.1 ± 0.1
6-^19^F-Trp-*Bs*CspB	316.6 ± 0.1
4-^19^F-Phe-*Bs*CspB (^19^F)	318.4 ± 0.2

The change in enthalpy of protein unfolding at *T*_M_, *ΔH*(*T*_M_), has been used as global parameter in Eq. (4) taking all seven ^1^H-detected folded-to-unfolding transitions into account and has been determined to *ΔH*(*T*_M_) = 197 ± 2 kJ/mol. For ^19^F detection, the change in enthalpy has been determined to *ΔH*(*T*_M_) = 176 ± 6 kJ/mol. The change in heat capacity has been fixed to *ΔC*_P_ = 5.8 kJ/(mol K) as it has been specifically determined for *Bs*CspB using NMR spectroscopy before relying on both heat and cold denaturing^45^.

The thermodynamic parameters calculated using thermal denaturation probed with NMR spectroscopy (Table 2) show excellent agreement with those derived by chemical denaturation monitored using intrinsic fluorescence (Table 1). Thus, 4-^19^F-Trp-*Bs*CspB shows a small increase in overall thermodynamic stability (approx. 4 Kelvin) compared to wild type *Bs*CspB whereas 2-^19^F-Phe-*Bs*CspB and 5-^19^F-Trp-*Bs*CspB variants show a small decrease or uniformity in Δ*G*° (Table 1) and *T*_M_ (Table 2), respectively.

### Unfolding and refolding kinetics probed by kinetic stopped-flow fluorescence

**Figure 4:**
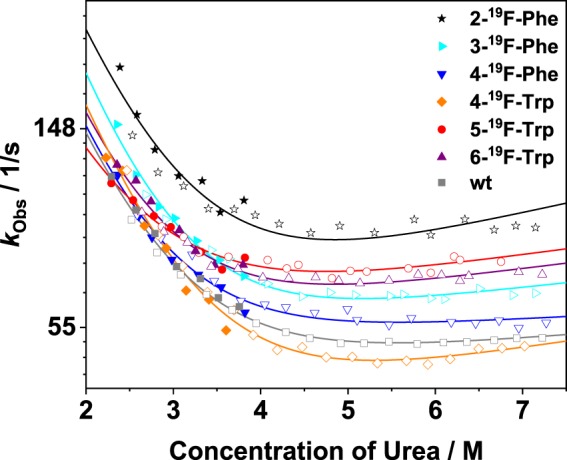
Dependence of the apparent rate constant on the concentration of urea characterizing unfolding (open symbols) and refolding (closed symbols) of fluorine-labelled variants of *Bs*CspB (stars represent 2-^19^F-Phe-, triangles with the tip to right represent 3-^19^F-Phe-, triangles with the tip to bottom represent 4-^19^F-Phe-, diamonds represent 4-^19^F-Trp-, circles represent 5-^19^F-Trp-, and triangles with the tip to top represent 6-^19^F-Trp-variant), and wild type protein (represented using rectangles). The straight lines represent best fits of Eq. (5) to the experimental data sharing the same value for the cooperativity of folding, *m* = *m*_f_ + *m*_u_. Results of this fitting procedure are summarized in Table 3.

The thermodynamic analysis of all six differently fluorine-labelled variants has been further extended to kinetic experiments using a stopped-flow fluorescence device. This setup enables to monitor the change in fluorescence on a millisecond time scale induced by rapid mixing of two solutions representing either folding or unfolding conditions^46^. A monoexponential function has been applied to obtain the apparent rate constant, *k*_obs_, to account for refolding (Fig. S18) and unfolding (Fig. S19) kinetics of fluorine-labelled variants and wild type *Bs*CspB. Having values for *k*_obs_ representing final concentrations of urea in the mixing cell ranging between *c*_urea_ = 2.3 M and *c*_urea_ = 7.3 M in hands, Eq. (5) has been applied to obtain rate constants for refolding, *k*_f_, and unfolding, *k*_u_, in absence of any denaturant, respectively. Moreover, Eq. (5) enables the determination of the slope of the unfolding, *m*_u_, and the refolding limb, *m*_f_, respectively. The fitting of Eq. (5) to the experimental data has been performed assuming a global value for the cooperativity of folding, *m* = *m*_f_ + *m*_u_, for all differently fluorine-labelled variants of *Bs*CspB including wild type protein. The Chevron plot analysis reveals a two-state folding process for all variants of *Bs*CspB which have been kinetically probed (Fig. 4). The cooperativity of folding has been determined to *m* = −2.8 ± 0.8 kJ/(mol M) matching the value of *m* = −2.9 ± 0.1 kJ/(mol M) which has been independently determined in the present study by monitoring the folded-to-unfolding transition of all variants of *Bs*CspB using equilibrium fluorescence (Fig. 2, Table 1). Comparing the kinetic rate constants for unfolding of fluorine-labelled variants of *Bs*CspB (31 s^−1^ ≤ *k*_u_ ≤ 57 s^−1^) does not reveal a significant difference to wild type *Bs*CspB (*k*_u_ = 40 ± 1 s^−1^, Table 3). We note that refolding kinetics for differently fluorine-labelled variants of *Bs*CspB using *c*_urea_ < 2.3 M could not be reliably acquired. However, obtaining refolding rate constants for all differently fluorine-labelled variants of *Bs*CspB by applying Eq. (5) enables a comparison among individual *k*_f_ values in a qualitative manner as all kinetic data have been analyzed in the same way. Thus, the refolding rate constants which have been determined for all fluorine-labelled variants of *Bs*CspB are slightly higher (except for 5-^19^F-Trp-*Bs*CspB) compared to *k*_f_ = 1050 ± 20 s^−1^ obtained for wild type *Bs*CspB (Table 3). The determination of the rate constants accounting for refolding und unfolding enables to apply Eq. (6) to compute the difference in free energy, Δ*G*°, of all variants of *Bs*CspB. As a result, these values are highly similar (Table 3) ranging between Δ*G*° = 6.4 ± 0.1 kJ/mol (5-^19^F-Trp-*Bs*CspB) and Δ*G*° = 9.5 ± 0.1 kJ/mol (4-^19^F-Trp-*Bs*CspB) covering the thermodynamic stability determined for wild type (Δ*G*° = 8.1 ± 0.1 kJ/mol). Comparing the values of Δ*G*° determined for all differently fluorine-labelled variants of *Bs*CspB by applying a kinetic setup with the overall thermodynamic stability obtained in equilibrium using chemical (Fig. 2, Table 1) or thermal denaturation (Fig. 3, Table 2) reveals two main features. Firstly, the overall thermodynamic stability of *Bs*CspB does not change significantly if fluorine has been attached either to all seven phenylalanine residues or to its single tryptophan residue. Secondly, it has been consistently shown by three independent experimental methods that 4-^19^F-Trp-*Bs*CspB exhibits a small increase in Δ*G*° by about 1 kJ/mol and in *T*_M_ by about 4 Kelvin whereas 5-^19^F-Trp-*Bs*CspB shows a moderate decrease in Δ*G*° by about 1.5 kJ/mol and a conserved value of *T*_M_ compared to wild type *Bs*CspB. Focusing on fluorine labelled phenylalanine variants, 4-^19^F-Phe-*Bs*CspB has a thermodynamic stability which is about 1 kJ/mol lower as the value for Δ*G*° which has been determined for 2-^19^F-Phe-*Bs*CspB and 3-^19^F-Phe-*Bs*CspB, respectively (Tables 1 and 3).

**Table 3:** Analysis of unfolding and refolding kinetics of wild type and fluorine-labelled variants of *Bs*CspB using *kinetic stopped-flow fluorescence*.

Protein	*k*_u_/s^−1^	*k*_f_/s^−1^	*m*_u_/kJ/(molM)	*m*_f_/kJ/(molM)	Δ*G*^0^/kJ/mol
wt *Bs*CspB	40 ± 1	1050 ± 20	0.1	−2.9	8.1 ± 0.1
2-^19^F-Phe-*Bs*CspB	56 ± 2	2000 ± 100	0.2	−3.0	8.9 ± 0.2
3-^19^F-Phe*-Bs*CspB	42 ± 1	1600 ± 40	0.2	−3.0	9.0 ± 0.1
4-^19^F-Phe-*Bs*CspB	44 ± 1	1050 ± 30	0.1	−2.9	7.9 ± 0.1
4-^19^F-Trp-*Bs*CspB	31 ± 1	1450 ± 30	0.2	−3.0	9.5 ± 0.1
5-^19^F-Trp-*Bs*CspB	57 ± 1	770 ± 20	0.1	−2.9	6.4 ± 0.1
6-^19^F-Trp-*Bs*CspB	48 ± 1	1160 ± 30	0.2	−3.0	7.9 ± 0.1

The cooperativity of folding, *m* = *m*_f_ + *m*_u_, has been used as global parameter in Eq. (5) taking all seven variants of *Bs*CspB into account and has been determined to *m* = −2.8 ± 0.8 kJ/(mol M). The value of *ΔG*^0^ has been computed by using Eq. (6).

We have extended the analysis of the kinetic data set to the information included in the amplitude of the monoexponential function following the refolding and unfolding of differently fluorine-labelled variants of *Bs*CspB. Thus, we have observed that the final values of the kinetics reporting on refolding and unfolding of fluorine-labelled variants do not converge as illustrated by 4-^19^F-Phe-*Bs*CspB (Fig. S20A,B). The refolding kinetics for all fluorine variants probed here show consistently lower amplitudes compared to unfolding kinetics (Fig. S20C–G). Note that the endpoint analysis performed for wild type *Bs*CspB based on kinetic stopped-flow data shows an unfolding transition which is anticipated (Fig. S20H). Consequently, we have examined the feature of non-converging endpoints seen for refolding and unfolding of fluorine-labelled *Bs*CspB variants by performing a set of control experiments. Firstly, 4-^19^F-Phe-*Bs*CspB has been mixed with denaturing buffer leading to *c*_urea_ = 2.6 M. Furthermore, unfolded 4-^19^F-Phe-*Bs*CspB present in *c*_urea_ = 7 M has been mixed with native buffer leading to *c*_urea_ = 2.6 M, too. The fluorescence emission of both unfolded and refolded 4-^19^F-Phe-*Bs*CspB has been immediately measured. As a result, the fluorescence intensity representing refolded 4-^19^F-Phe-*Bs*CspB is lower compared to the fluorescence intensity observed for unfolded 4-^19^F-Phe-*Bs*CspB (Fig. S21A). Quantitatively, the fluorescence intensity monitoring unfolding of 4-^19^F-Phe-BsCspB is about 30% higher compared to the fluorescence intensity monitored for protein refolding (Fig. S21A). The higher fluorescence intensity observed for unfolding of 4-^19^F-Phe-*Bs*CspB compared to refolding matches the difference seen in fluorescence amplitude observing folding kinetics of 4-^19^F-Phe-*Bs*CspB independently acquired at the stopped-flow instrument (Fig. S20A). Secondly, potential slow unfolding or refolding of 4-^19^F-Phe-*Bs*CspB taking place on a second-to-minute time scale cannot be observed as the fluorescence emission spectra monitored directly after mixing and after 30 minutes look identical (Fig. S21A). Thirdly, monitoring refolding and unfolding of wild type *Bs*CspB by using identical experimental conditions reveals a different result. Both refolding and unfolding of wild type *Bs*CspB observed in *c*_urea_ = 2.6 M lead to almost identical profiles in fluorescence emission intensity (Fig. S22). Fourthly, we have applied high-resolution NMR spectroscopy to further unravel the origin of the gap present in amplitude analysis of the kinetic data observed for fluorine-labelled BsCspB variants (Fig. S21B). Thus, the reversibility of folded-to-unfolding reaction of 4-^19^F-Phe-*Bs*CspB has been quantitatively probed by acquiring one-dimensional ^1^H NMR data on both unfolded and refolded samples, respectively (Fig. S21B). The fraction of native 4-^19^F-Phe-*Bs*CspB, *f*_N_, can be determined by calculating the ratio *f*_N_ = *I*_N_/(*I*_N_ + *I*_N+U_) using integrals representing native, *I*_N_, as native and unfolded, *I*_N+U_, signals of *Bs*CspB. The analysis of both the unfolding and the refolding reaction of 4-^19^F-Phe-*Bs*CspB leads to almost identical values for *f*_N_, namely *f*_N_ = 0.191 and *f*_N_ = 0.189, respectively. This result shows that 4-^19^F-Phe-*Bs*CspB folds fully reversibly and that there is no apparent long-term folding rate constant additionally present which may explain the gap observed in the amplitude analysis of the kinetic data. Taken these results together, we conclude that adding singly fluorinated tryptophan or phenylalanines to *Bs*CspB inherently changes the properties in tryptophan fluorescence emission of this protein. This change in fluorescence emission can be observed when comparing fluorine-labelled *Bs*CspB which has been refolded with the unfolded counterpart. Contrary, applying high-resolution NMR spectroscopy does not reveal structural differences in fluorine-labelled *Bs*CspB when comparing refolded with unfolded protein which has been modified by fluorotryptophan or fluorophenylalanines.

### Structure determination of 4-^19^F-Phe-*Bs*CspB and 4-^19^F-Trp-*Bs*CspB by X-ray crystallography

**Figure 5:**
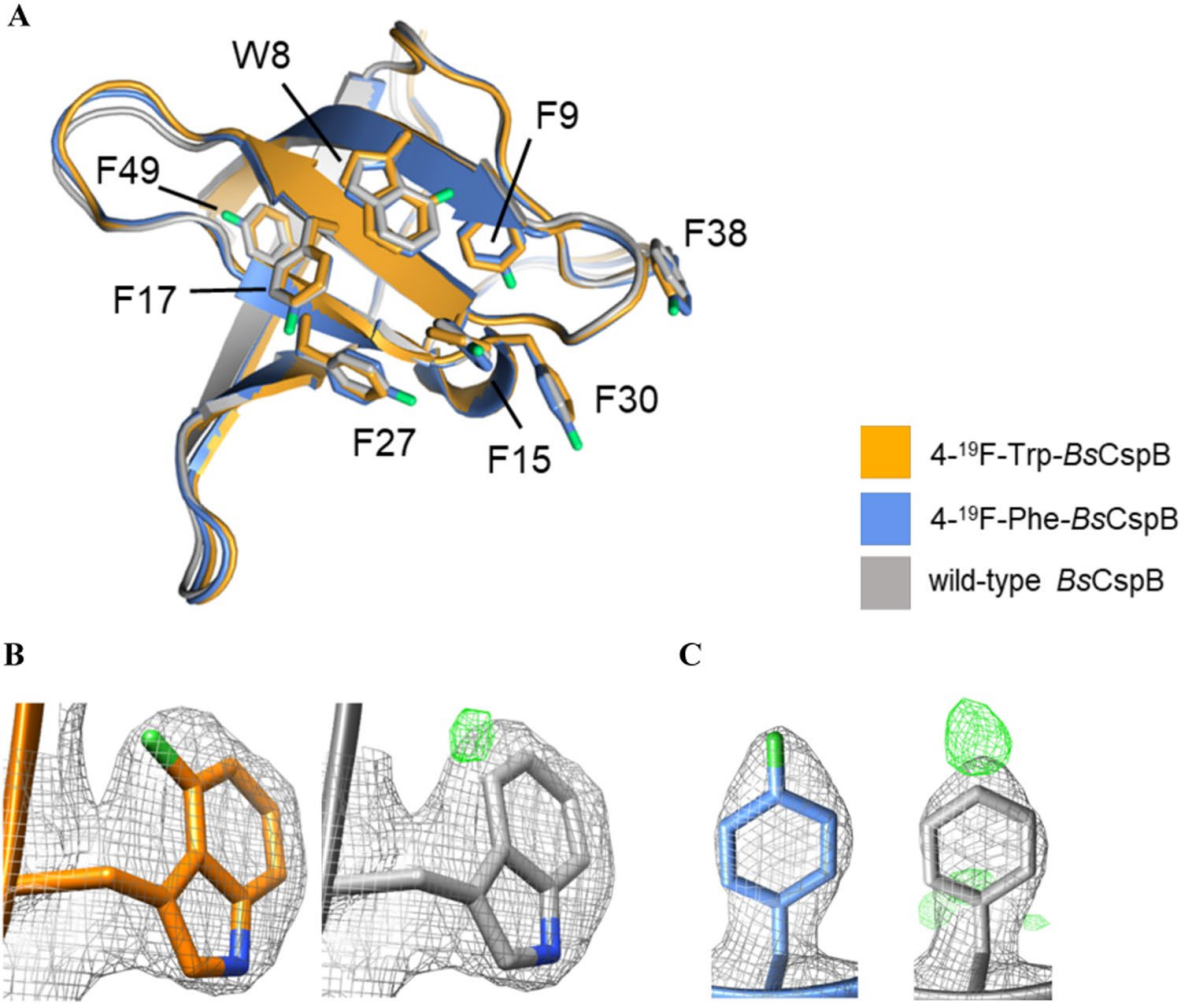
Three-dimensional structures of ^19^F-derivatized *Bs*CspB. (**A**) The crystal structures of 4-^19^F-Trp*-Bs*CspB (colored in orange) and 4-^19^F-Phe*-Bs*CspB (colored in blue) are shown superimposed onto wild type *Bs*CspB (pdb code 1NMG, colored in grey). The side chains of ^19^F-derivatized residues in this study are displayed and labelled according to their sequence numbering. (**B**,**C**) Electron density maps of residues ^19^F-Trp8 (B) and ^19^F-Phe49 (as representative) are shown. (2mF_obs_ − DF_calc_)α_calc_ maps contoured at 1σ are colored in grey. (mF_obs_ − DF_calc_) maps calculated using phases from refined models with omitted ^19^F-derivatized residues are shown in green color, contoured at 3σ. The structures have been created by using Chimera^59^, version 1.14 (www.cgl.ucsf.edu/chimera).

Probing fluorine-labelled variants of *Bs*CspB using fluorescence and NMR spectroscopy has independently shown that 4-^19^F-Trp-*Bs*CspB has a slightly increased thermodynamic stability, whereas Δ*G*° of 4-^19^F-Phe-*Bs*CspB is slightly decreased compared to wild type protein. To investigate potential structural changes in 4-^19^F-Trp-*Bs*CspB and 4-^19^F-Phe-*Bs*CspB, we have elucidated their atomic structures using X-ray crystallography to 2.05 Å and 2.1 Å resolution, respectively (Fig. 5, Table S2). The samples crystallized in a closely related crystallographic lattice, containing one single molecular copy of modified *Bs*CspB in the asymmetric unit and sharing a same space group symmetry. The global RMSD for all C_α_ atoms for the 4-^19^F-Trp-*Bs*CspB and 4-^19^F-Phe-*Bs*CspB structures calculated in this work is 0.23 Å, indicating that the fold trace of the two variants is virtually identical. A comparison of the variants here studied with a previously reported crystal structure of wild type *Bs*CspB elucidated in a different crystallographic symmetry (PDB ID: 1CSP) revealed RMSD_Cα_ values of 0.52 Å and 0.37 Å for 4-^19^F-Trp-*Bs*CspB and 4-^19^F-Phe-*Bs*CspB, respectively (Fig. 5). The excellent agreement across all structures confirmed that ^19^F derivatization did not induce any detectable alterations of the fold. Furthermore, wild type and ^19^F-derivative structures displayed identical conformational rotamers for the modified Trp and Phe residues, indicating that local structural distortions had also not taken place (Fig. 5). When comparing wild type and derivatives, the RMSD values for all atoms in the modified residues were: 0.48 Å for residue Trp8, 0.35 Å for Phe9, 0.30 Å for Phe15, 0.15 Å for Phe17, 0.43 Å for Phe27, 0.2 Å for Phe30, 0.65 Å for Phe38, and 0.3 Å for Phe49. Based on this excellent agreement, we conclude that the introduction of the ^19^F-labels in *Bs*CspB did not induce structural changes of significance even when seven residues were modified.

## Conclusion

### Synergistic combination of equilibrium and kinetic fluorescence with NMR spectroscopy and X-ray crystallography

We have presented an integrated approach to structurally and dynamically characterize fluorine-labelled proteins using a repertoire of orthogonal biophysical methodologies. Fluorescence experiments conducted in equilibrium following the folded-to-unfolding reaction of fluorine-labelled *Bs*CspB induced by chemical denaturation as well as monitoring the refolding and unfolding of these samples by fluorescence emission in a time-resolved manner elucidated only small differences in overall thermodynamic stability. This conservation in thermodynamic stability regarding wild type behaviour could be confirmed by monitoring the thermal denaturation of fluorine-labelled *Bs*CspB using one-dimensional ^1^H and ^19^F NMR spectroscopy. The crystallization and structure determination of fluorine modified *Bs*CspB corroborated the findings presented above by showing an almost perfect overlay between wild type and ^19^F-Phe or ^19^F-Trp modified protein variants taking both backbone and side chain atoms into account. To our knowledge, this represents the first three-dimensional structure determination of a fluorophenylalanine-labelled protein possessing more than a single fluorination site which has been reported so far. Note that the solution structure of a fluorinated side chain labelled villin headpiece subdomain comprising 35 residues has been presented before^31^. Thus, the coherent characterization of fluorine-labelled *Bs*CspB done here consistently show only mild effects protein labelling causes on structural and dynamic properties by using singly fluorinated amino acids, even when multiple sites in the protein have been used for modification. The integrated approach for the determination of the impact fluorine-labelling has on proteins converges to the main finding. In fact, incorporation of singly fluorinated Trp or Phe into *Bs*CspB induces, if at all, only slight changes in structural and dynamic parameters. Interestingly, these slight changes are independent of the number and sites of fluorine atoms which have been inserted into *Bs*CspB: one fluorine atom used for ^19^F-Trp labelling (sequence position 8) or seven fluorine atoms used for ^19^F-Phe labelling (sequence positions 9, 15, 17, 27, 30, 38, and 49). This modest impact of protein labelling using fluorine has been partially presented for other proteins but only by using either a limited number of experimental techniques or a reduced number of different fluorinated amino acids which have been used for protein labelling. In this regard, a combination of CD and NMR spectroscopy showed that the three-dimensional structure and thermodynamic stability of GB1 protein is not significantly affected when 5-^19^F-Trp has been used for labelling^47^. Moreover, the structure of 5-^19^F-Trp- and 6-^19^F-Trp-labelled annexin V has been determined by X-ray crystallography^48^ elucidating only minimal changes in the local protein geometry compared to non-labelled protein and slight changes in thermal melting observed by CD spectroscopy which are on the same order as elucidated here for fluorine-labelled *Bs*CspB. Similarly, the crystal structure of 5-^19^F-Trp-labelled triosephosphate isomerase showed no discrepancies in local and global structural properties comparing labelled with non-labelled protein^49^, the same property has been reported for anthrax protective antigen indicating that 5-^19^F-Trp-labelling minimally perturb structural properties seen for wild type protein^50^ or for fluoroacetate dehalogenase utilizing also 5-^19^F-Trp-labelling^22^. Specialized studies have focused e.g. on relaxation properties of fluorinated amino acids in free and protein-bound form^51^, general NMR parameters of isolated fluorine-labelled amino acids^52^ or the application of homonuclear ^19^F-^19^F EXSY NMR on a fluorine-labelled receptor protein^53^. We are aware that we have solely probed one protein in this study. However, the experimental design has been done following a highly systematic strategy by incorporating six different fluorinated amino acids into *Bs*CspB. Such fluorine-labelled variants of *Bs*CspB have been subsequently probed applying various orthogonal biophysical techniques which led to a consistent result. For this reason, we believe that the present study closes an important gap in the characterization of fluorine-labelled proteins by obtaining a convergent view on the impact that the insertion of fluorine into proteins has. We propose that single fluorine-labelled tryptophan and phenylalanine residues may serve as ideal candidates for the incorporation into proteins enabling experiments to understand protein performance in highly biological contexts like cell lysates or even *in cellula*.

## Methods

### Protein expression and purification

The six variants of the cold shock proteins B from *Bacillus subtilis* differing in the position of the fluorine (2-^19^F-Phe-, 3-^19^F-Phe-, 4-^19^F-Phe-, 4-^19^F-Trp-, 5-^19^F-Trp-, and 6-^19^F-Trp-*Bs*CpB, Figs. 1 and S1) were expressed in *E. coli* cells by using pET24a CspB and pAR1219 vectors as described previously^32^ (DSMZ 12779 strain was used for the three Phe variants whereas the strain CAG 18455 7371 was used for the three Trp variants). Subsequently, an established protocol for protein purification was applied^32^. The concentration, *c*, of the purified proteins was determined by measuring the absorbance at λ = 280 nm, *A*_280_, of the protein solution in a *d* = 1 cm long cuvette in an UV/Vis spectrometer (Agilent 8453 UV-visible Spectroscopy System, Agilent Technologies) employing extinction coefficients of *ε*^°280^ = 2705 M^−1^  cm^−1^ (4-^19^F-Trp *Bs*CspB), *ε*^280^ = 2887 M^−1^  cm^−1^ (5-^19^F-Trp *Bs*CspB), *ε*^280^ = 2575 M^−1^  cm^−1^ (6-^19^F-Trp *Bs*CspB), and *ε*^280^ = 5800 M^−1^  cm^−1^ (2-^19^F-Phe *Bs*CspB, 3-^19^F-Phe *Bs*CspB, 4-^19^F-Phe *Bs*CspB, and wild type *Bs*CspB)^32^. The Lambert Beer law, *A*_280_ = c*d*ε^280^, was then applied to the determination of *c*. Fluorine labelling efficiency can be specified with >95%^32^.

### Fluorescence spectroscopy in steady state

Fluorescence experiments performed in steady state were conducted on a FP-8500 Spectrofluorometer (Jasco). The final concentration of the protein was set to *c* = 1 µM in all experiments. Samples were thermally equilibrated for at least 30 minutes and measured under stirring condition at a temperature *T* = 298 K, using 20 mM sodiumcacodylate, pH 7.0. The individual folded-to-unfolding transitions of *Bs*CspB variants were monitored between *c* = 0 M and *c* = 8 M urea applying 34 data points. Fluorescence spectra were acquired as triplicates in the wavelength (*λ*) range 290 nm to 400 nm in steps of 0.5 nm by using a wavelength for excitation of *λ* = 280 nm. The concentration of urea, *c*_urea,_ was determined by measuring the refractive index (Eq. (2))^54^.

2 $${c}_{{\rm{urea}}}=117.66\ast \varDelta n+29.753\ast \varDelta {n}^{2}+185.56\ast \varDelta {n}^{3},$$

where *Δn* reflects the difference in the refractive index of the buffer solution taking absence and presence of urea into account.

All equilibrium protein folded-to-unfolding transitions were background subtracted and measured in duplicate.

The Eq. (3) was applied to determine the difference in free energy between the folded and the unfolded state of *Bs*CspB, *ΔG*^0^, and the cooperativity of protein unfolding, *m*

3 $$\langle \lambda \rangle =\frac{({g}_{{\rm{N}}}+{m}_{{\rm{N}}}\ast {c}_{{\rm{u}}{\rm{r}}{\rm{e}}{\rm{a}}})+({g}_{{\rm{U}}}+{m}_{{\rm{U}}}\ast {c}_{{\rm{u}}{\rm{r}}{\rm{e}}{\rm{a}}})\ast (\exp (\,-\,\frac{\varDelta {G}^{0}}{RT})+\frac{m\ast {c}_{{\rm{u}}{\rm{r}}{\rm{e}}{\rm{a}}}}{RT})}{1+(\exp (\,-\,\frac{\varDelta {G}^{0}}{RT})+\frac{m\ast {c}_{{\rm{u}}{\rm{r}}{\rm{e}}{\rm{a}}}}{RT})},$$

where $\langle \lambda \rangle$ represents the intensity averaged emission wavelength^41^, *g*_N/U_ and *m*_N/U_ account for the baselines of the folded and the unfolded state, *R* is the universal gas constant, *c*_urea_ is the concentration of urea, and *T* is the absolute temperature^42^.

### NMR spectroscopy

All one-dimensional ^1^H NMR spectra were acquired at protein concentrations ranging between *c* = 200 µM and *c* = 650 µM and measured in 20 mM sodiumcacodylate containing 90% H_2_O and 10% D_2_O (*v*/*v*) at pH = 7.0. NMR data were collected on an 800 MHz Bruker Avance NEO NMR spectrometer equipped with a TCI cryogenically cooled probe possessing a proton channel which allows tuning of the ^19^F resonance at *ω*_L_^19F^ = 753 MHz. The proton resonance frequency of trimethylsilylproanoic acid (TMSP) was used for direct referencing of all ^1^H spectra. One-dimensional ^19^F NMR spectra were indirectly referenced by using the information obtained for referencing of protons. The processing of NMR data used TOPSPIN 4.0.3 software (Bruker Biospin, Germany).

The determination of the thermodynamic stability of the different variants of *Bs*CspB was monitored by one-dimensional ^1^H- and ^19^F- NMR spectroscopy (holds for 4-^19^F-Phe-*Bs*CspB) at different temperatures ranging between *T* = 291 K and *T* = 330 K. Two ranges differing in chemical shifts were used for the determination of the fraction of unfolded protein, *f*_U_, being present at different temperatures. The first range, *I*_N_, covers chemical shifts between 0.59 ppm and 0.14 ppm representing signals seen for the folded state. The second range, *I*_N+U_, covers chemical shifts between 0.697 ppm and 1.064 ppm representing signals indicating both the folded and the unfolded state, respectively. Using the ratio *I*_N_/(*I*_N_ + *I*_N+U_) enables the precise determination of the total population of the folded state, *f*_N_, at any temperature^45^. The population of the unfolded state, *f*_U_, can now be determined assuming a two-state folding scenario as described for *Bs*CspB, *f*_U_ = 1 − *f*_N_^33^. The fraction of unfolded protein, *f*_U_, has been subsequently used to determine the temperature midpoint, *T*_M_, of the folded-to-unfolding transition of different variants of *Bs*CspB by using Eq. (4)

4 $${f}_{{\rm{U}}}(T)=\frac{\exp (-\frac{\varDelta {H}_{{\rm{U}}}({T}_{{\rm{M}}})(\frac{{T}_{{\rm{M}}}-T}{{T}_{{\rm{M}}}})-\varDelta {C}_{{\rm{P}}}({T}_{{\rm{M}}}-T+T\ast \,\mathrm{ln}(\frac{T}{{T}_{{\rm{M}}}}))}{RT})}{1+\exp (-\frac{\varDelta {H}_{{\rm{U}}}({T}_{{\rm{M}}})(\frac{{T}_{{\rm{M}}}-T}{{T}_{{\rm{M}}}})-\varDelta {C}_{{\rm{P}}}({T}_{{\rm{M}}}-T+T\ast \,\mathrm{ln}(\frac{T}{{T}_{{\rm{M}}}}))}{RT})},$$

where *ΔH*_U_ represents the van’t Hoff enthalpy of unfolding at *T*_M_, *ΔC*_P_ the change in heat capacity between folded und unfolded state, *R* is the universal gas constant, and *T* is the absolute temperature^45^.

### Kinetic measurements using fluorescence stopped-flow methodology

Kinetic fluorescence spectroscopic data were collected by using a SX20 Stopped Flow Spectrometer (Applied Photophysics, UK). After excitation at a wavelength of 280 nm the folding kinetics was recorded by the change of fluorescence above a wavelength of 320 nm using a cut off filter. All single mixing experiments were performed in 20 mM sodiumcacodylate at pH = 7.0 and *T* = 298 K. The protein solution of *c* = 15 µM present in the native buffer was diluted 11-fold with urea solutions (in 20 mM sodiumcacodylate, pH = 7.0) of different concentrations leading to final concentrations of urea in the measuring cell ranging between *c*_urea_ = 2.6 M and 7.3 M to detect kinetics of protein unfolding. Refolding kinetics of unfolded protein of *c* = 15 µM present in *c*_urea_ = 7 M and 20 mM sodiumcacodylate, pH = 7.0 were followed by an 11-fold dilution with urea solutions (in 20 mM sodiumcacodylate, pH = 7.0) at different concentrations, leading to final concentrations of urea in the measuring cell ranging between *c*_urea_ = 0.6 M and 3.9 M. All folding and refolding kinetics were measured 12 times under identical conditions and averaged. Data processing used the Pro Data Viewer software (Applied Photophysics, UK).

Kinetic data were analysed using a monoexponential function. The apparent rate constant, *k*_obs_, has been plotted logarithmically as a function of *c*_urea_. Data analysis applying Eq. (5) enabled the determination of the rate constants of protein refolding *k*_f_ and unfolding *k*_u_, respectively, assuming a two state folding process

5 $$\mathrm{ln}({k}_{{\rm{obs}}})=\exp (\mathrm{ln}({k}_{{\rm{f}}})+\frac{{m}_{{\rm{f}}}\ast {c}_{{\rm{urea}}}}{RT})+\exp (\mathrm{ln}({k}_{{\rm{u}}})+\frac{{m}_{{\rm{u}}}\ast {c}_{{\rm{urea}}}}{RT}).$$

Here, *m*_f_ and *m*_u_ represent the limbs for protein refolding and unfolding leading to the cooperativity of folding *m* = *m*_f_ + *m*_u_, which was independently determined before using Eq. (3), *R* is the universal gas constant, and *T* is the absolute temperature^33^.

Finally, the rate constants for refolding, *k*_f_, and unfolding *k*_u_, can be used to determine the overall thermodynamic stability, Δ*G*^0^, of the protein under investigation. Thus the difference in free energy of a protein sensing folding or unfolding conditions can be obtained by using Eq. (6)

6 $$\varDelta {G}^{0}=-\,RT\ast ln\,K=-\,RT\ast ln\frac{[U]}{[N]}=-\,RT\ast ln\frac{{k}_{{\rm{u}}}}{{k}_{{\rm{f}}}},$$

where *K* is the equilibrium constant, *R* is the universal gas constant, and *T* is the absolute temperature^33^.

### X-ray crystallography

Protein samples 4-^19^F-Phe*-Bs*CspB and 4-^19^F-Trp*-Bs*CspB were concentrated to 20 mg/ml in 20 mM sodium cacodylate pH 7.0 and 100 mM NaCl and crystallized using a Gryphon liquid dispenser (Art Robbins instruments) on 96-well Intelli-plates (Art Robbins instruments) employing the sitting drop method at 18 °C. Crystals of 4-^19^F-Phe-*Bs*CspB grew from 100 mM CHES pH 9.5, 1 M sodium citrate tribasic. Crystals of 4-^19^F-Trp-*Bs*CspB grew from 0.1 M BIS-TRIS propane pH 7.0, 1.2 M sodium citrate tribasic dehydrate. Drop consisted of a 150 nl:150 nl protein:precipitate ratio. For X-ray data cryo-collection, crystals were vitrified in LN_2_ in mother liquor supplemented with 25% [v/v] glycerol. X-ray diffraction data were collected at the Swiss Light Source synchrotron (Villigen) and processed using XDS/XSCALE^55^. Phasing was by molecular replacement in PHASER^56^ using the crystal structure of wild type CspB (PDB ID: 1CSP) as search model. Manual model building was in COOT^57^ and refinement used PHENIX.refine^58^. Chemical libraries for the modified residues 4-^19^F-Phe (PFF) and 4-^19^F-Trp (4FW) were obtained from the Protein Data Bank. Visual structure comparisons and calculations of root-mean-square deviations (RMSD) were performed in Chimera^59^.

## Supplementary information

Supporting Information.
