# Supplementary material for: What does fluorine do to a protein? Thermodynamic, and highly-resolved structural insights into fluorine-labelled variants of the cold shock protein
Source: Sci Rep. 2020 Feb 14;10:2640. doi: 10.1038/s41598-020-59446-w (PMC7021800; doi:10.1038/s41598-020-59446-w)
Supplement: Supplementary file 1 — Supporting Information. [file 41598_2020_59446_MOESM1_ESM.pdf]

What does fluorine do to a protein? Thermodynamic, and highly-resolved structural insights into fluorine-labelled variants of the cold shock protein

Hannah Welte[1,2], Tiankun Zhou[3], Xenia Mihajlenko[1], Olga Mayans[2,3] and Michael Kovermann[1,2,4*]

[1] Department of Chemistry, Universitätsstrasse 10, Universität Konstanz, DE-78457 Konstanz.

[2] Graduate School Chemical Biology KoRS-CB, Universitätsstrasse 10, Universität Konstanz, DE-78457 Konstanz.

[3] Department of Biology, Universitätsstrasse 10, Universität Konstanz, DE-78457 Konstanz.

[4] Zukunftskolleg, Universitätsstrasse 10, Universität Konstanz, DE-78457 Konstanz.

*correspondence: michael.kovermann@uni-konstanz.de

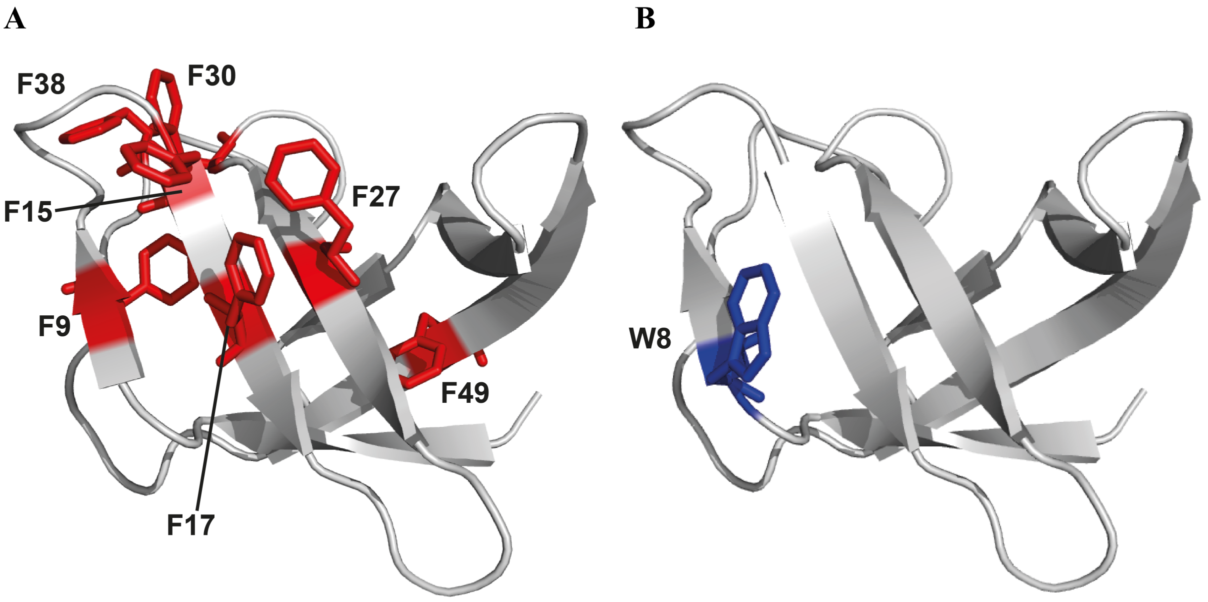

Figure S1

Three-dimensional presentation of wild type *Bs*CspB highlighting the seven intrinsic phenylalanine (A, colored in red) and the single tryptophan residue (B, colored in blue). Note that all seven phenylalanines have been substituted by using 2-^{19}F-Phe, 3-^{19}F-Phe or 4-^{19}F-Phe to compose 2-^{19}F-Phe-, 3-^{19}F-Phe- or 4-^{19}F-Phe-*Bs*CspB. Replacing W8 by 4-^{19}F-Trp, 5-^{19}F-Trp or 6-^{19}F-Trp leads to ^{19}F-Trp-, 5-^{19}F-Trp- or 6-^{19}F-Trp-*Bs*CspB. The pdb code 1NMG has been used to prepare this figure. The structures have been created by using the PyMOL Molecular Graphics System, Version 2.4a0, Schrödinger, LLC (www.pymol.org).

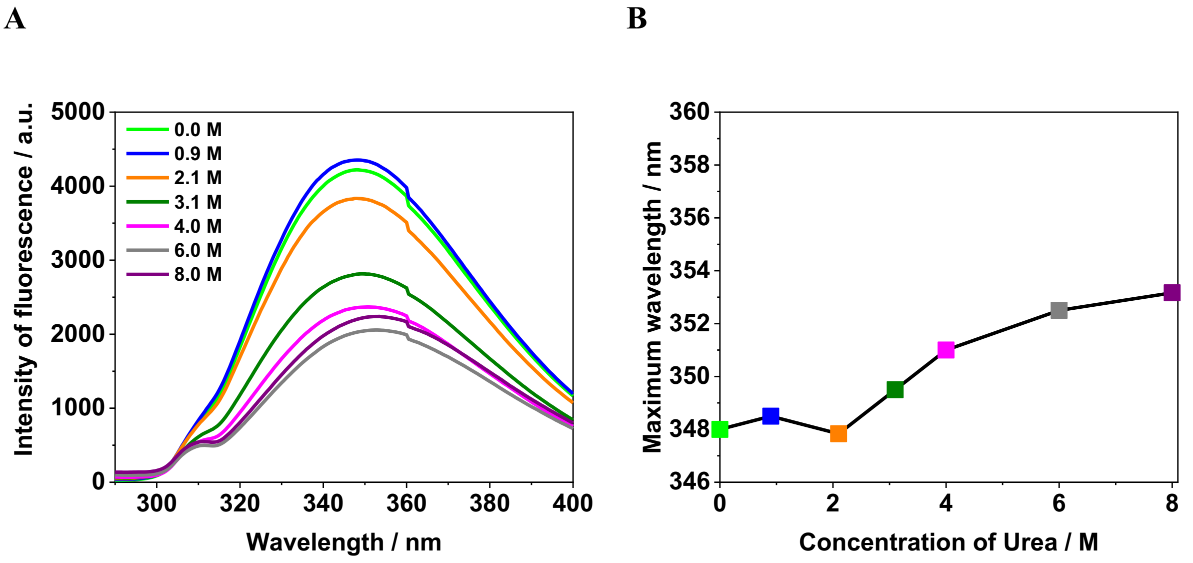

Figure S2

(A) Fluorescence spectra for wild type *Bs*CspB obtained at different concentrations of urea by using a wavelength of excitation of λ = 280 nm. (B) Maximum in emission wavelength obtained at different concentrations of urea for *Bs*CspB.

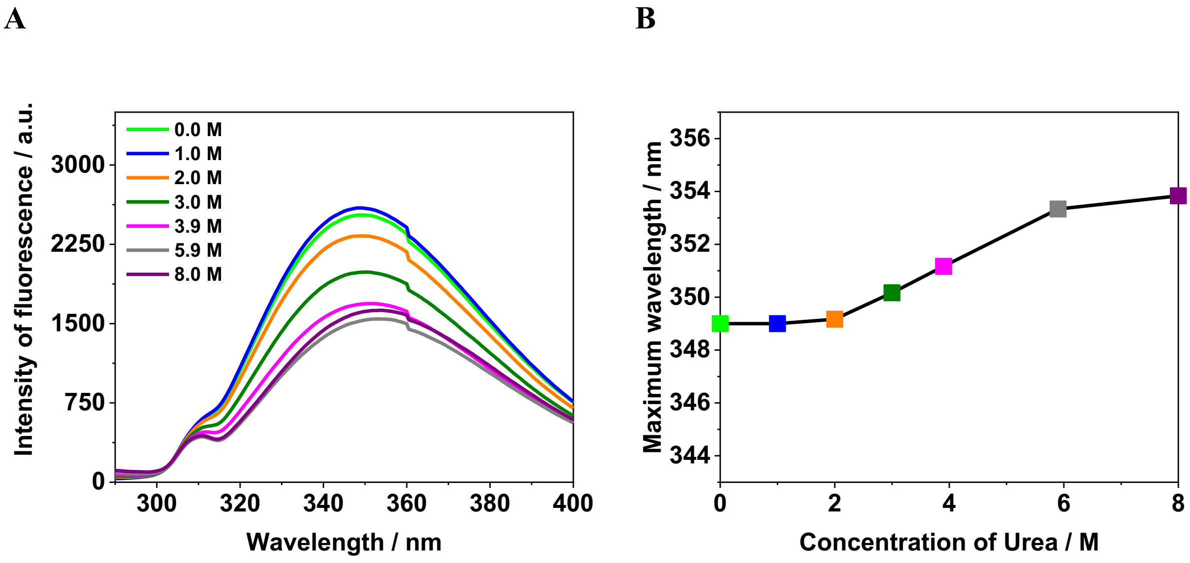

Figure S3

(A) Fluorescence spectra for 2-^{19}F-Phe-*Bs*CspB obtained at different concentrations of urea by using a wavelength of excitation of λ = 280 nm. (B) Maximum in emission wavelength obtained at different concentrations of urea for 2-^{19}F-Phe-*Bs*CspB.

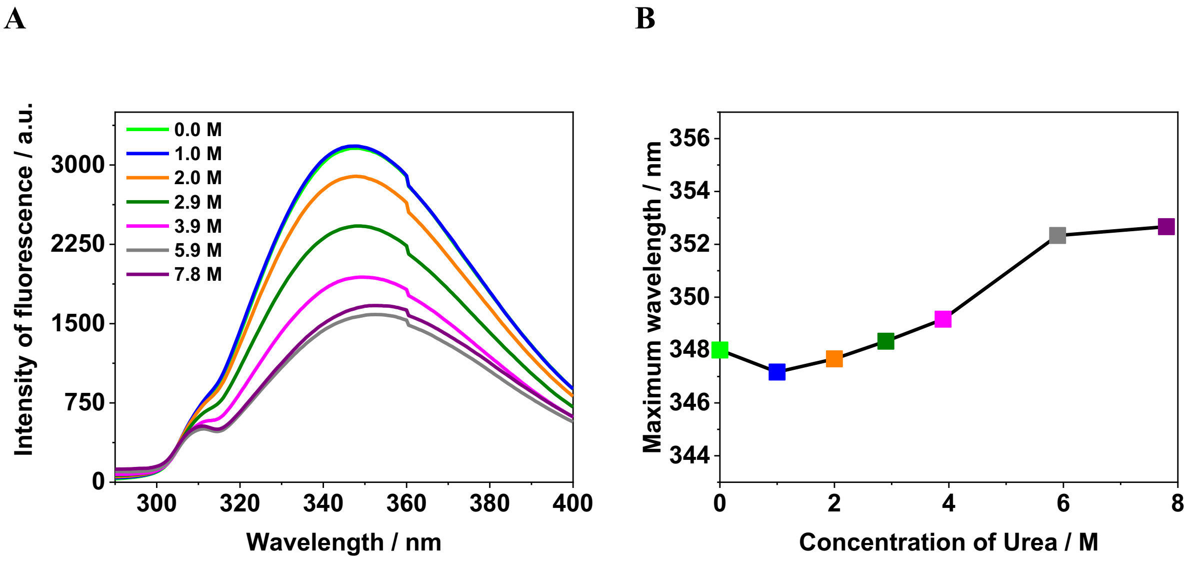

Figure S4

(A) Fluorescence spectra for 3-^{19}F-Phe-*Bs*CspB obtained at different concentrations of urea by using a wavelength of excitation of λ = 280 nm. (B) Maximum in emission wavelength obtained at different concentrations of urea for 3-^{19}F-Phe-*Bs*CspB.

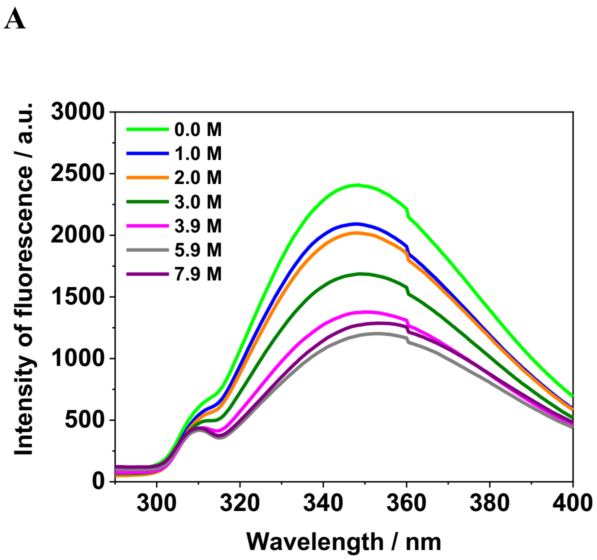

Figure S5

(A) Fluorescence spectra for 4-^{19}F-Phe-*Bs*CspB obtained at different concentrations of urea by using a wavelength of excitation of λ = 280 nm. (B) Maximum in emission wavelength obtained at different concentrations of urea for 4-^{19}F-Phe-*Bs*CspB.

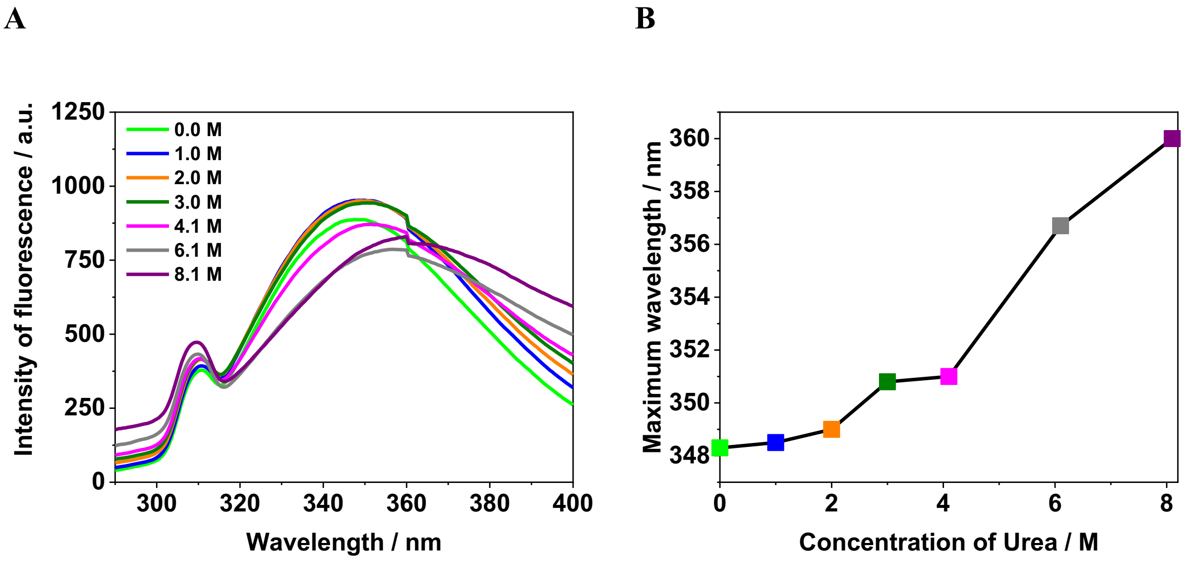

Figure S6

(A) Fluorescence spectra for 4-^{19}F-Trp-*Bs*CspB obtained at different concentrations of urea by using a wavelength of excitation of λ = 280 nm. (B) Maximum in emission wavelength obtained at different concentrations of urea for 4-^{19}F-Trp-*Bs*CspB.

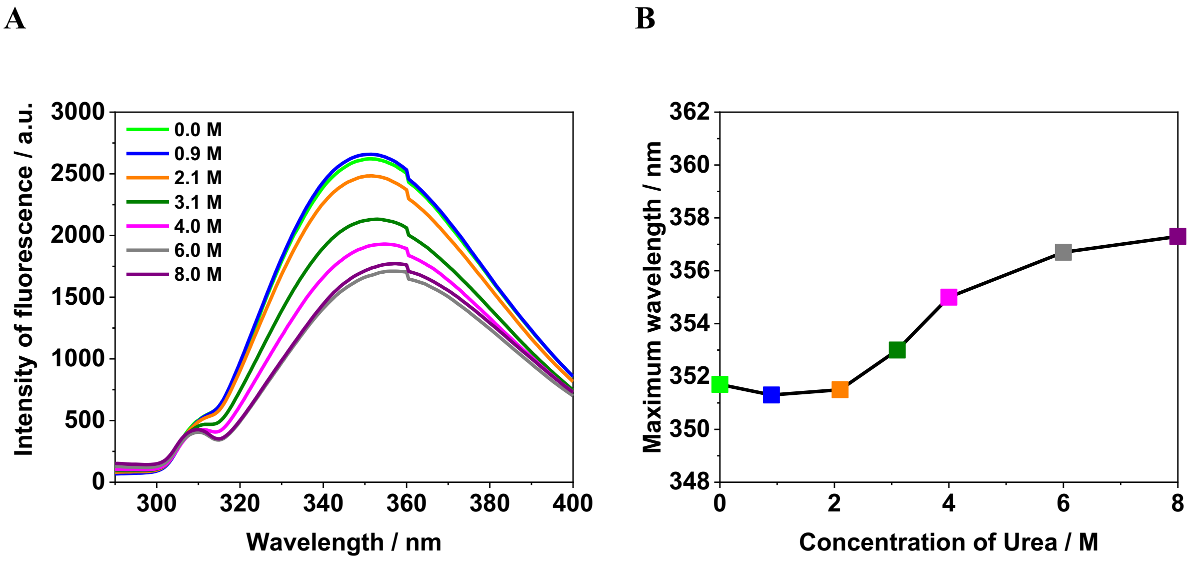

Figure S7

(A) Fluorescence spectra for 5-^{19}F-Trp-*Bs*CspB obtained at different concentrations of urea by using a wavelength of excitation of λ = 280 nm. (B) Maximum in emission wavelength obtained at different concentrations of urea for 5-^{19}F-Trp-*Bs*CspB.

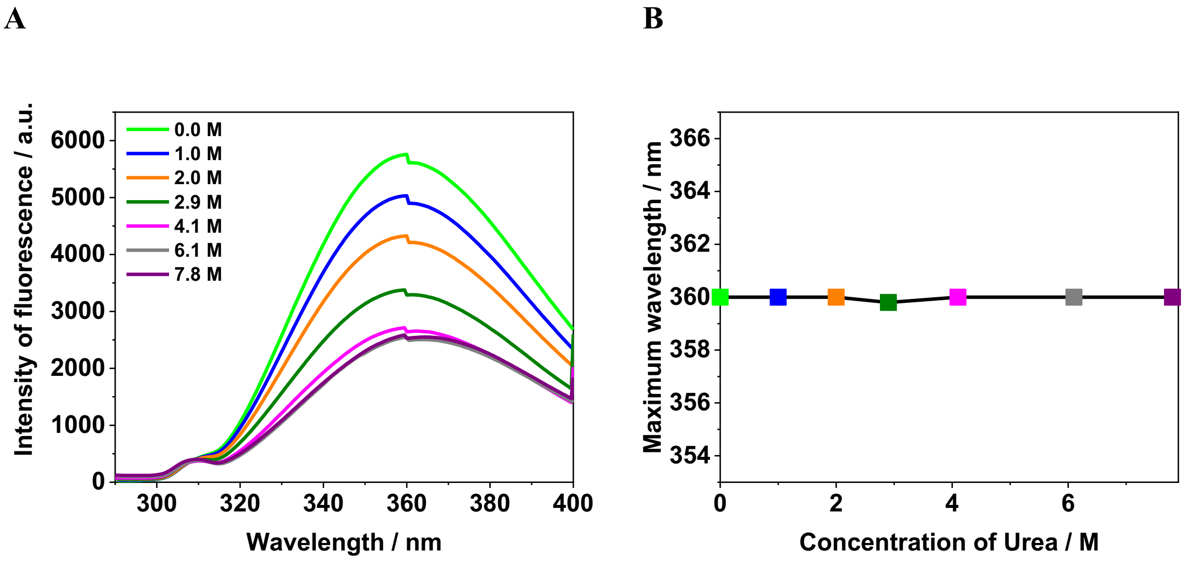

Figure S8

(A) Fluorescence spectra for 6-^{19}F-Trp-*Bs*CspB obtained at different concentrations of urea by using a wavelength of excitation of λ = 280 nm. (B) Maximum in emission wavelength obtained at different concentrations of urea for 6-^{19}F-Trp-*Bs*CspB.

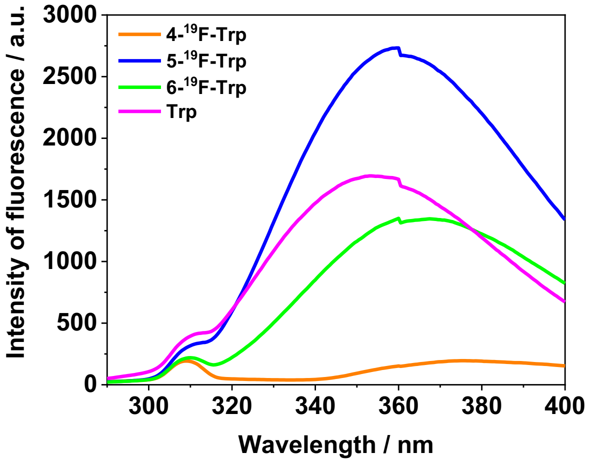

Figure S9

Fluorescence emission spectra of the free, single amino acid residues tryptophan (colored in pink), 4-^{19}F-tryptophan (colored in orange), 5-^{19}F-tryptophan (colored in blue) and 6-^{19}F-tryptophan (colored in green). A concentration of c = 1 μM has been used for the acquisition of all four fluorescence emission spectra shown here. The wavelength for excitation has been set to 280 nm, the temperature to T = 298 K. All single amino acids have been dissolved in 20 mM sodiumcacodylate, pH 7.0 without precipitation.

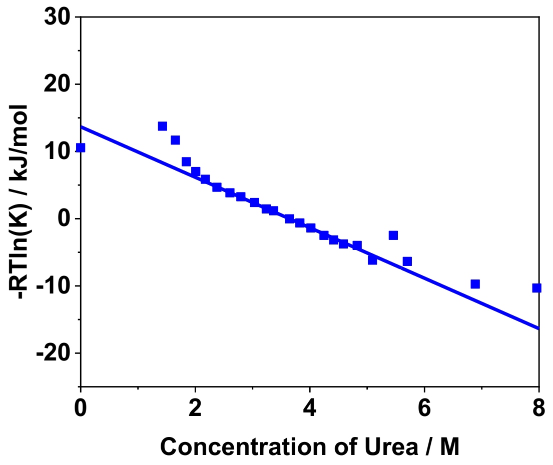

Figure S10

Linear fit of the natural logarithm of the equilibrium constant acquired for 4-^{19}F-Phe-*Bs*CspB to determine ΔG^0 and m according to equations (S1) and (S2). For linear fitting, 2.4 M $\leq c^{urea} \leq$ 4.8 M has been used. Results are shown in Table S1.

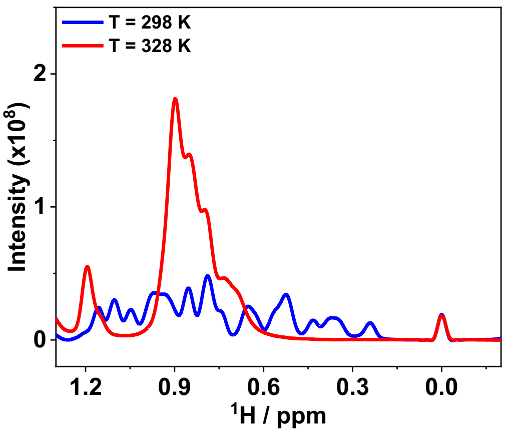

Figure S11

One-dimensional proton NMR spectra of wild type *Bs*CspB representing aliphatic protons acquired at $T = 298$ K (colored in blue) and $T = 328$ K (colored in red).

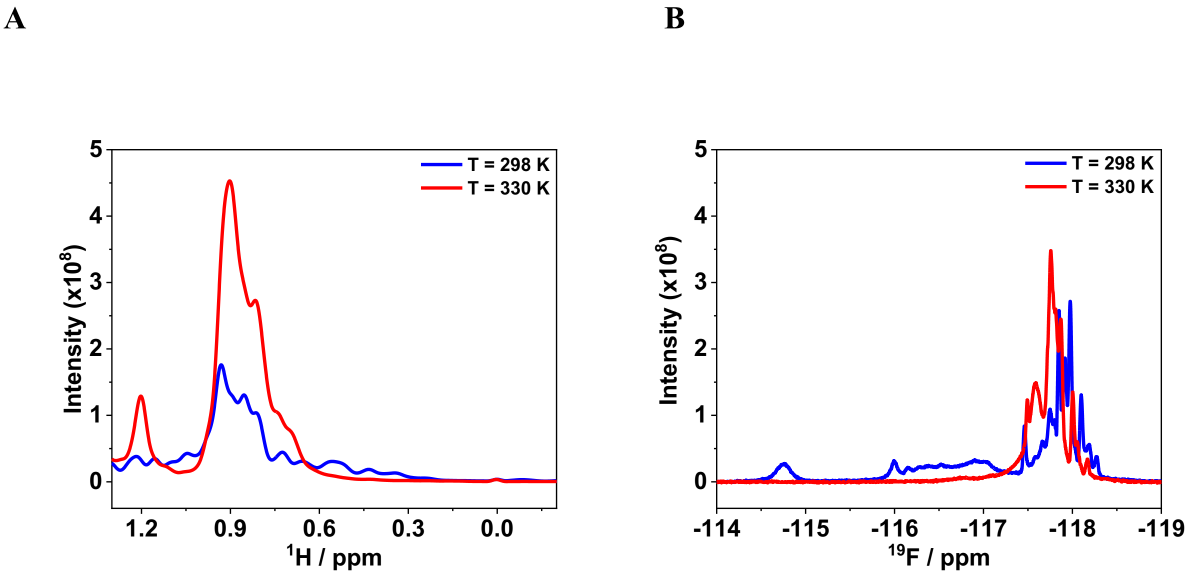

Figure S12

One-dimensional NMR spectra of 2-^{19}F-Phe-*Bs*CspB representing aliphatic protons (A) and fluorine resonance signals (B) acquired at T = 298 K (colored in blue) and T = 330 K (colored in red).

A

B

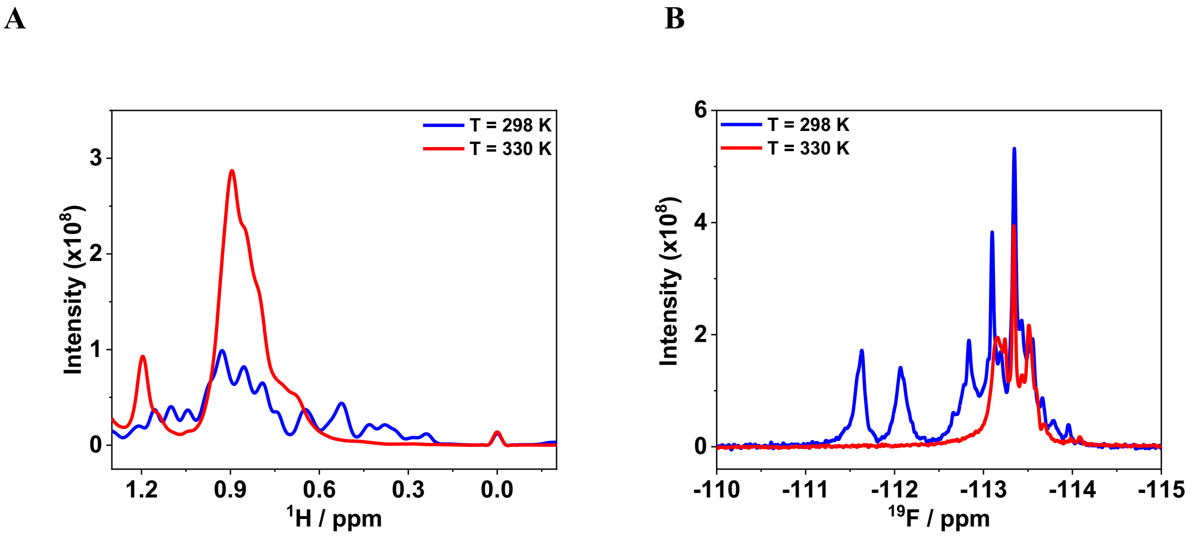

Figure S13

One-dimensional NMR spectra of 3-^{19}F-Phe-*Bs*CspB representing aliphatic protons (A) and fluorine resonance signals (B) acquired at $T = 298$ K (colored in blue) and $T = 330$ K (colored in red).

A

B

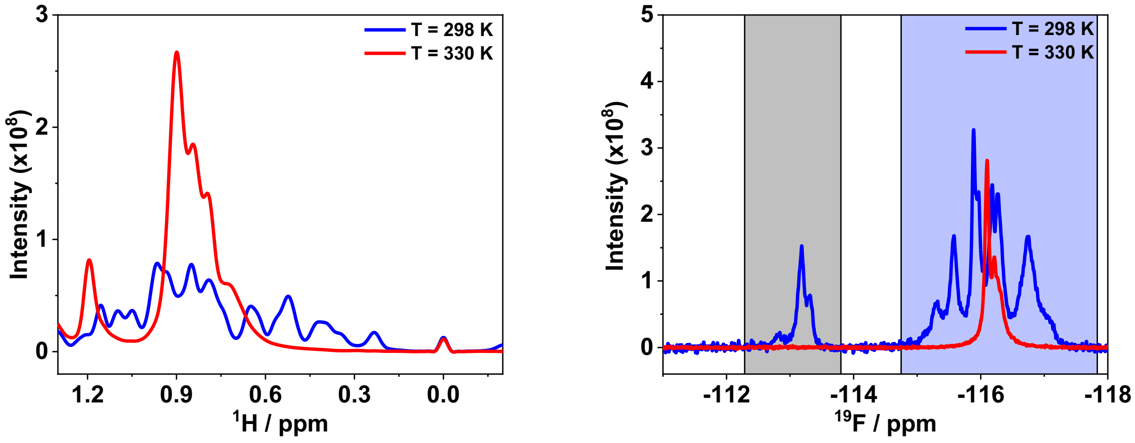

Figure S14

One-dimensional NMR spectra of 4-^{19}F-Phe-*Bs*CspB representing aliphatic protons (A) and fluorine resonance signals (B) acquired at T = 298 K (colored in blue) and T = 330 K (colored in red). For the analysis of the thermodynamic stability of 4-^{19}F-Phe-*Bs*CspB using fluorine spectra, two ranges of chemical shifts have been used. The range colored in gray indicate resonance signals originating from the native state (I_N) whereas the range of chemical shifts colored in light blue represents resonance signals originating from both, the native state as well as the unfolded protein ensemble (I_{N+U}). Using the ratio $I_N/(I_N+I_{N+U})$ enables the precise determination of the total population of the folded state, f_N, at any temperature.

A B

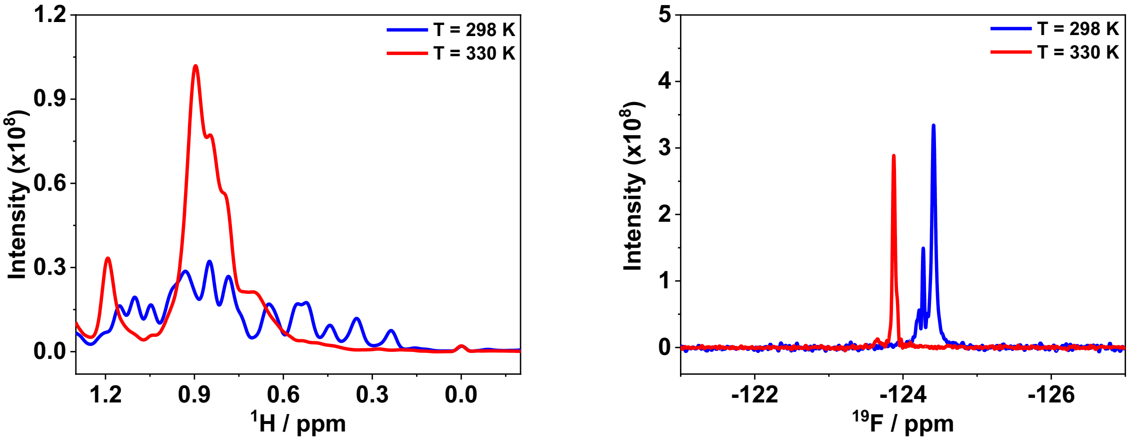

Figure S15

One-dimensional NMR spectra of 4-^{19}F-Trp-*Bs*CspB representing aliphatic protons (A) and fluorine resonance signals (B) acquired at T = 298 K (colored in blue) and T = 330 K (colored in red).

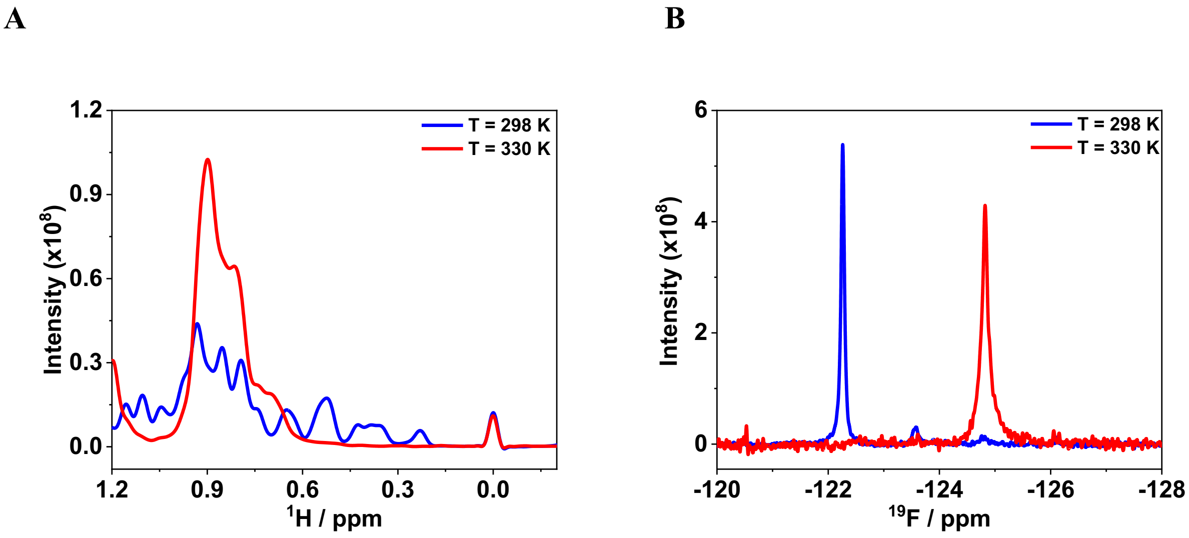

Figure S16

One-dimensional NMR spectra of 5-^{19}F-Trp-*Bs*CspB representing aliphatic protons (A) and fluorine resonance signals (B) acquired at $T = 298$ K (colored in blue) and $T = 330$ K (colored in red).

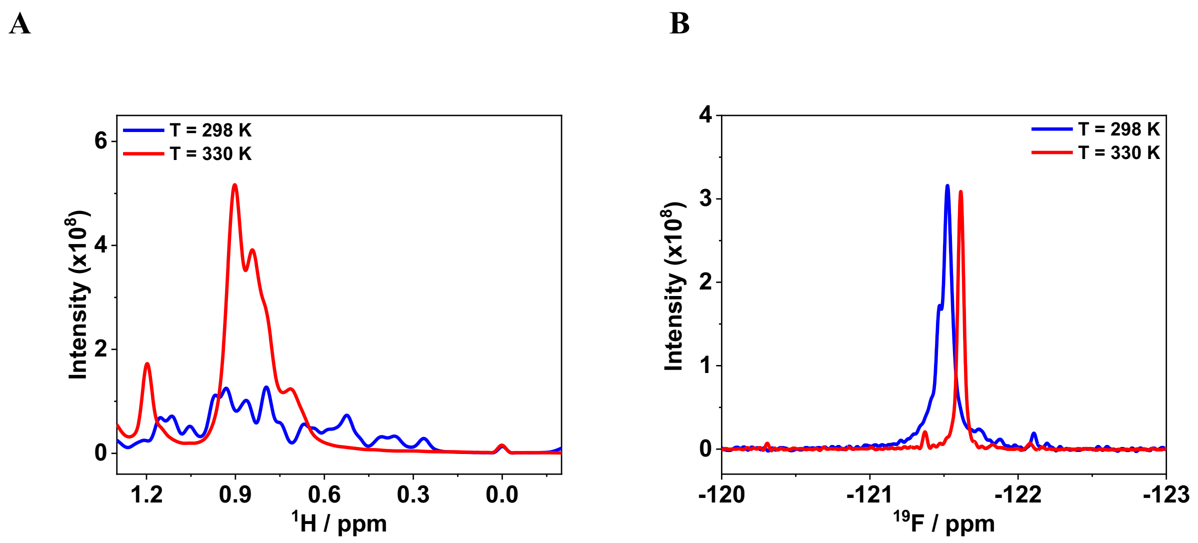

Figure S17

One-dimensional NMR spectra of 6-^{19}F-Trp-*Bs*CspB representing aliphatic protons (A) and fluorine resonance signals (B) acquired at T = 298 K (colored in blue) and T = 330 K (colored in red).

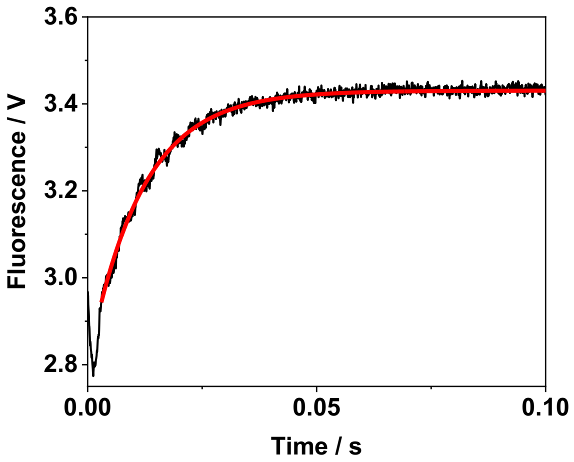

Figure S18

Refolding kinetics of 4-^{19}F-Phe-*Bs*CspB initiated by a change in buffer conditions following c = 7.0 M urea to c = 3.0 M urea. A mono-exponential function was used for the range covering $9 \leq t \leq 60$ ms for data fitting: $y(t) = a*\exp(-k*t)+c$. The coefficients have been determined to $c = 3.430 \pm 0.001$, $a = -0.630 \pm 0.006$ and $k = (85.8 \pm 0.8)$ s^{-1}.

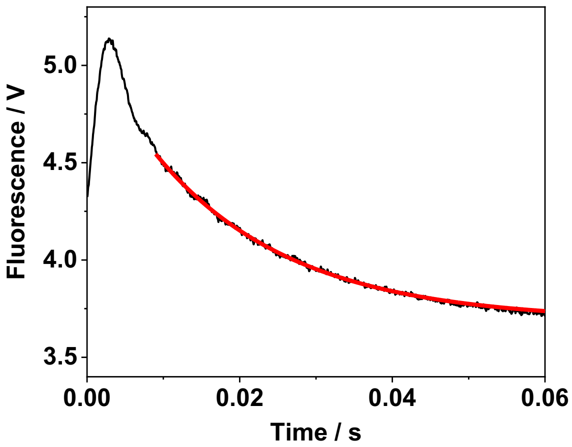

Figure S19

Unfolding kinetics of 4-^{19}F-Phe-*Bs*CspB initiated by a change in buffer conditions following c = 0.5 M to c = 6.5 M urea. A mono-exponential function was used for the range covering $10 \leq t \leq 40$ ms for data fitting: $y(t) = a{*}\exp(-k{*}t)+c$. The coefficients have been determined to $c = 3.687 \pm 0.002$, $a = 1.415 \pm 0.004$ and $k = (55.7\pm0.4)$ s^{-1}.

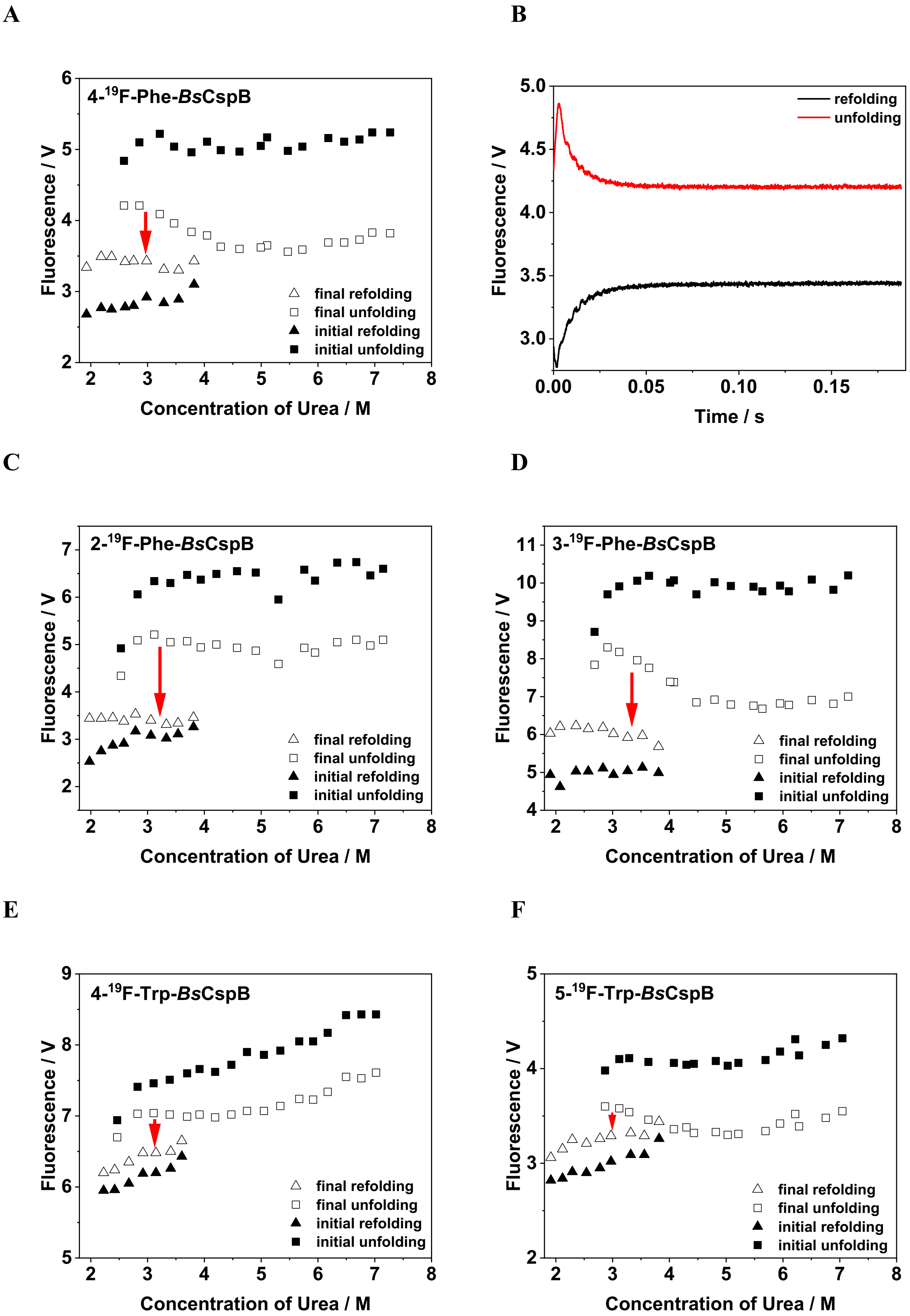
A
4-^{19}F-Phe-*Bs*CspB
Fluorescence / V
Concentration of Urea / M
final refolding
final unfolding
initial refolding
initial unfolding
B
Fluorescence / V
Time / s
refolding
unfolding
C
2-^{19}F-Phe-*Bs*CspB
Fluorescence / V
Concentration of Urea / M
final refolding
final unfolding
initial refolding
initial unfolding
D
3-^{19}F-Phe-*Bs*CspB
Fluorescence / V
Concentration of Urea / M
final refolding
final unfolding
initial refolding
initial unfolding
E
4-^{19}F-Trp-*Bs*CspB
Fluorescence / V
Concentration of Urea / M
final refolding
final unfolding
initial refolding
initial unfolding
F
5-^{19}F-Trp-*Bs*CspB
Fluorescence / V
Concentration of Urea / M
final refolding
final unfolding
initial refolding
initial unfolding

G

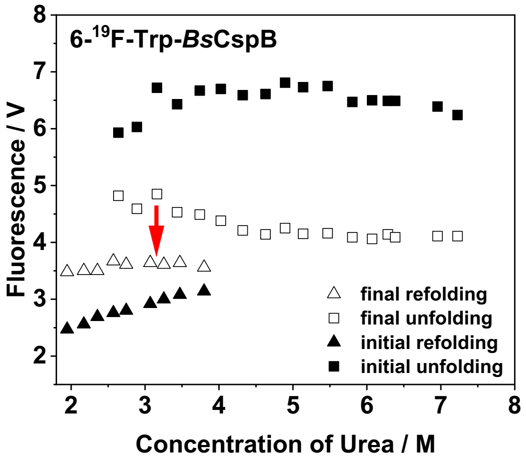

H

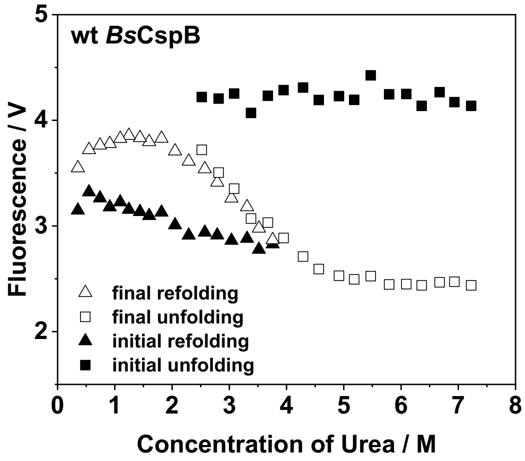

Figure S20

Analysis of amplitudes of time-dependent changes in fluorescence emission following unfolding (square) and refolding (triangle) of fluorine labelled variants of *Bs*CspB (A) 4-^{19}F-Phe-*Bs*CspB, (C) 2-^{19}F-Phe-*Bs*CspB, (D) 3-^{19}F-Phe-*Bs*CspB, (E) 4-^{19}F-Trp-*Bs*CspB, (F) 5-^{19}F-Trp-*Bs*CspB, (G) 6-^{19}F-Trp-*Bs*CspB and (H) wild type *Bs*CspB. Open symbols show the final fluorescence value and closed symbols the initial fluorescence value. The arrow colored in red shown in (A), (C)-(G) indicates the gap observed in fluorescence emission when comparing refolding with unfolding condition. (B) Time-dependent change in fluorescence emission for the refolding (colored in black) and unfolding (colored in red) of 4-^{19}F-Phe-*Bs*CspB monitored at the stopped-flow fluorescence spectrometer representing a final concentration of c = 2.6 M urea.

A **B**

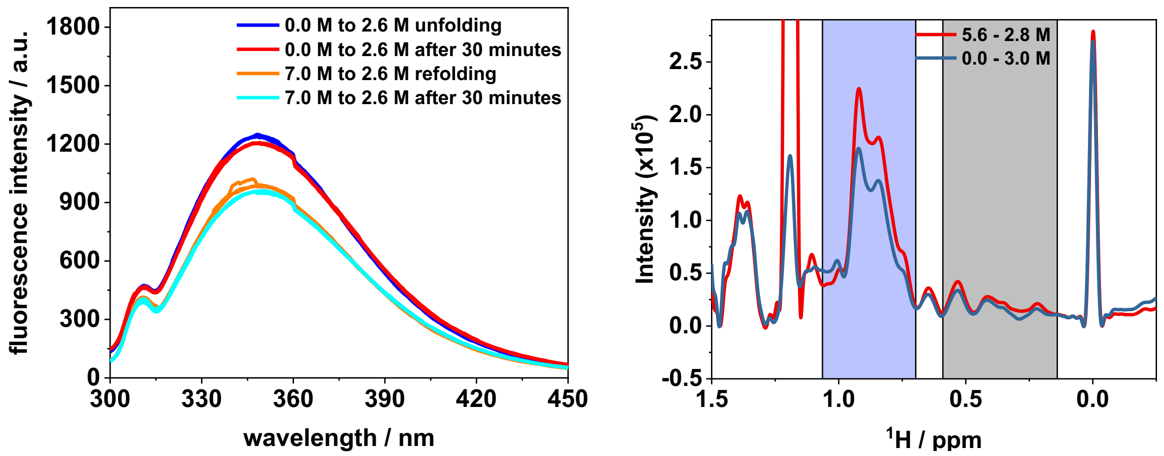

Figure S21

Analysis of protein refolding and unfolding using fluorescence (A) and NMR spectroscopy (B). Fluorescence spectra have been acquired at the Spectrofluorometer for 4-^{19}F-Phe-*Bs*CspB (c = 1 µM) directly (colored in blue) and 30 minutes after initiating protein unfolding (colored in red), respectively, using a wavelength of excitation of λ = 280 nm in a solution containing c = 2.6 M urea. Protein refolding of 4-^{19}F-Phe-*Bs*CspB (c = 1 µM) has been acquired directly (colored in orange) and after 30 minutes after mixing (colored in cyan), respectively, using a wavelength of excitation of λ = 280 nm in a solution containing c = 2.6 M urea. One-dimensional ^{1}H NMR spectra have been acquired for 4-^{19}F-Phe-*Bs*CspB (c = 20 µM) after unfolding of a solution containing c = 3.0 M urea (colored in blue) and refolding containing c = 2.8 M (colored in red), respectively. For the analysis of the fraction of the native state, two ranges of chemical shifts of the one-dimensional ^{1}H NMR spectrum of 4-^{19}F-Phe-*Bs*CspB have been used for integration. The range colored in gray indicate resonance signals originating from the native state whereas the range of chemical shifts colored in light blue represents resonance signals originating from both, the native state as well as the unfolded protein ensemble, respectively.

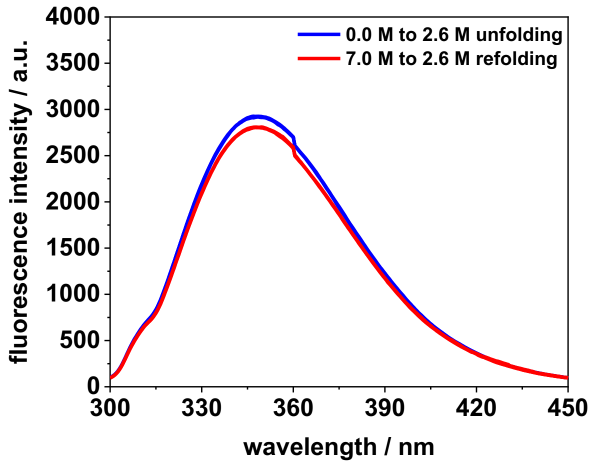

Figure S22

Analysis of refolding (colored in red) and unfolding (colored in blue) of wild type *Bs*CspB (c = 1 µM) using fluorescence spectroscopy. Spectra of fluorescence emission have been acquired using a wavelength of excitation of λ = 280 nm in a solution containing c = 2.6 M urea.

Table S1

Individual analysis of equilibrium unfolding of wild type and two replicates of fluorine labeled *Bs*CspB using *fluorescence spectroscopy* to determine the overall thermodynamic stability, ΔG^0. The cooperativity of protein unfolding has been used as global parameter in equation (3) taking all seven folded-to-unfolding transitions into account and has been determined to $m = -2.9 \pm 0.1$ kJ/(mol M). The midpoint of folded-to-unfolding transition, C_M, has been calculated using $C_M = \Delta G^0/m$. Note that data analysis has been performed by applying equation (3) and by linear extrapolation[1] using equations (S1) and (S2) illuminating the same value for m applying global analysis of this parameter. Data for ΔG^0 and C_M shown in Tab. 1 are based on the average of the two replicates analyzing the data using equation (3).

	ΔG^0 / kJ/mol	C_M / M	ΔG^0 / kJ/mol	C_M / M
	using eq. (S1), (S2)		using eq. (3)	
wt *Bs*CspB	11.6 ± 0.2	4.0 ± 0.1	11.1 ± 0.5	3.8 ± 0.2
2-^{19}F-Phe-*Bs*CspB	10.1 ± 0.1	3.5 ± 0.1	9.6 ± 0.7	3.3 ± 0.3
	10.6 ± 0.2	3.7 ± 0.1	10.5 ± 0.6	3.6 ± 0.2
3-^{19}F-Phe-*Bs*CspB	13.3 ± 0.1	4.6 ± 0.1	9.8 ± 0.7	3.4 ± 0.2
	12.4 ± 0.1	4.3 ± 0.1	11.2 ± 0.5	3.9 ± 0.2
4-^{19}F-Phe-*Bs*CspB	10.7 ± 0.2	3.7 ± 0.1	9.6 ± 0.6	3.3 ± 0.2
	11.1 ± 0.1	3.8 ± 0.1	9.6 ± 0.6	3.3 ± 0.2
4-^{19}F-Trp-*Bs*CspB	16.0 ± 0.1	5.5 ± 0.1	13.8 ± 1.1	4.8 ± 0.4
	12.2 ± 0.1	4.2 ± 0.1	11.6 ± 1.1	4.0 ± 0.4
5-^{19}F-Trp-*Bs*CspB	9.9 ± 0.3	3.4 ± 0.1	9.9 ± 0.5	3.4 ± 0.2
	9.5 ± 0.1	3.3 ± 0.1	8.2 ± 0.5	2.8 ± 0.2
6-^{19}F-Trp-*Bs*CspB	12.1 ± 0.1	4.2 ± 0.1	11.7 ± 0.7	4.0 ± 0.2
	11.1 ± 0.3	3.8 ± 0.1	10.4 ± 0.7	3.6 ± 0.2

Table S2

X-ray data collection and refinement statistics.

	4-^{19}F-Trp-*Bs*CspB	4-^{19}F-Phe-*Bs*CspB
**PDB**	6SZZ	6T00
**X-ray data**		
X-ray source	SLS-PXIII	SLS-PXI
Detector	PILATUS 2M-F	EIGER 16M X
Wavelength (Å)	1.000	1.000
**Data Collection**		
Space group	$P4_32_12$	$P4_32_12$
Cell dimensions		
a, b, c (Å)	56.05, 56.05, 55.12	54.94, 54.94, 57.71
α, β, γ (°)	90, 90, 90	90, 90, 90
Resolution (Å)	39.64-2.05 (2.15-2.05)	39.79-2.1 (2.2-2.1)
Copies in asymmetric unit	1	1
Solvent content (%)	58.15	58.4
Unique reflections	5913 (762)	5551 (692)
Multiplicity	24.75 (24.29)	12.43 (12.9)
Completeness (%)	99.8 (100)	99.9 (100)
$\langle I/\sigma(I)\rangle$	28.89 (1.25)	17.23 (1.23)
$R_{sym}(I)$ (%)	7.5 (273.5)	8.7 (225.2)
$CC_{1/2}$ (%)	100 (58.4)	99.9 (42.9)
**Model Refinement**		
AU content		
Protein/solvent non-H atoms	518/30	519/36
Ligands[a]	GOL x 2	NHE x 1, GOL x 1
R_{work}/R_{free}[b] (%)	20.83/23.12	19.87/25.07
RMSD bond length (Å)	0.008	0.01
RMSD bond angle (°)	0.922	1.251

[a]GOL: glycerol; NHE: CHES (*N*-Cyclohexyl-2-aminoethanesulfonic acid); [b]R-free sets corresponded to 5% of total reflections.

Analyzing emission of fluorescence dependent on the concentration of urea

Another method to obtain the overall thermodynamic stability of a protein lies in fitting the transition region of the denaturation curve linearly[1]. For a two-state process the Free Energy *ΔG* can be calculated for different concentrations of urea by using

$$\Delta G = -RT * \ln K \qquad \text{(S1)}$$

with the equilibrium constant

$$K = \frac{<\lambda> - (a_{\mathrm{N}} + n_{\mathrm{N}} * c_{\mathrm{urea}})}{(\mathrm{a}_{\mathrm{U}} + n_{\mathrm{U}} * c_{\mathrm{urea}}) - <\lambda>} \qquad \text{(S2)}$$

with $a_{\mathrm{N}} + n_{\mathrm{N}} * c_{\mathrm{urea}}$ representing a linear function for the baseline of the native state and $a_{\mathrm{U}} + n_{\mathrm{U}} * c_{\mathrm{urea}}$ representing a linear function for the baseline of the unfolded ensemble, respectively. Plotting *ΔG* dependent on the concentration of urea in the range of the folded-to-unfolding transition results in Gibb´s Free Energy, ΔG^0, in absence of urea representing the intersection with the y-axis and the cooperativity of folding, *m,* as the slope of the line.

1 Alexander, S. S. & Pace, C. N. Comparison of Denaturation of Bovine Beta-Lactoglobulins-a and B and Goat Beta-Lactoglobulin. *Biochemistry-Us* **10**, 2738-+ (1971).
